# Effect of 40 Hz light flicker on cognitive impairment and transcriptome of hippocampus in right unilateral common carotid artery occlusion mice

**DOI:** 10.1038/s41598-023-48897-6

**Published:** 2023-12-04

**Authors:** Zhaorui Niu, Minjie Yu, Peixia Xu, Renchuan Liu, Shangda Li, Congchong Wu, Bochao Huang, Xinyi Ye, Jianbo Hu, Yi Xu, Shaojia Lu

**Affiliations:** 1 Department of Psychiatry, The First Affiliated Hospital, Zhejiang University School of Medicine, No. 79 Qingchun Road, Hangzhou, 310003 Zhejiang China; 2 The Key Laboratory of Mental Disorder Management in Zhejiang Province, Hangzhou, 310003 China; 3 Brain Research Institute of Zhejiang University, Hangzhou, 310003 China; 4 Zhejiang Engineering Center for Mathematical Mental Health, Hangzhou, 310003 China; 5 Zhejiang Xinyue Health Consulting Service Medical Institution, Hangzhou, 310003 China

**Keywords:** Neuroscience, Diseases

## Abstract

Vascular cognitive impairment caused by chronic cerebral hypoperfusion (CCH) seriously affects the quality of life of elderly patients. However, there is no effective treatment to control this disease. This study investigated the potential neuroprotective effect of the 40 Hz light flicker in a mouse model of CCH. CCH was induced in male C57 mice by right unilateral common carotid artery occlusion (rUCCAO), leading to chronic brain injury. The mice underwent 40 Hz light flicker stimulation for 30 days after surgery. The results showed that 40 Hz light flicker treatment ameliorated memory deficits after rUCCAO and alleviated the damage to neurons in the frontal lobe and hippocampus. Light flicker administration at 40 Hz decreased IL-1β and TNF-α levels in the frontal lobe and hippocampus, but immunohistochemistry showed that it did not induce angiogenesis in mice with rUCCAO. Gene expression profiling revealed that the induction of genes was mainly enriched in inflammatory-related pathways. Our findings demonstrate that 40 Hz light flicker can suppress cognitive impairment caused by rUCCAO and that this effect may be involved in the attenuation of neuroinflammation.

## Introduction

Vascular cognitive impairment (VCI) is cognitive loss caused by vascular pathology, ranging from subjective cognitive impairment to severe dementia. It is the second most common cause of dementia and has caused enormous economic and social burdens in recent years^1^. To date, there are no treatments authorized by the American Food and Drug Administration or the European Medicines Agency to control the progression of this disease^2^. Therefore, it is urgent to develop more effective ways to cure patients.

While the mechanisms contributing to VCI are not completely understood, chronic age-dependent cerebral hypoperfusion is the most common underlying mechanism^3,4^. Cerebral hypoperfusion is a major cause of degenerative processes that lead to cognitive decline, and is associated with the severity of dementia^5,6^. To date, right unilateral common carotid artery occlusion (rUCCAO) is generally used to model chronic cerebral hypoperfusion in mice^7,8^. After rUCCAO, cerebral perfusion is reduced by 35% compared to baseline, and hypoperfusion in the ipsilateral hemisphere can last for 4 months^9,10^. In addition to a decrease in blood flow, rUCCAO has been shown to cause a variety of memory deficits in behavioral tests^11^, accompanied by neuronal degeneration and brain atrophy, which is similar to the neurodegenerative pathology observed in VCI patients^12,13^. Previous studies have reported that CCH is associated with neuroinflammation^14^. Ischemia induces the secretion of various cytokines, such as IL-1β and TNF-α^15,16^. The activation of inflammatory cytokines aggravates CCH injury. In addition, inhibiting neuroinflammation can relieve cognitive impairment in VCI mice^17^.

Gamma oscillations are rhythmic fluctuations of electrical activity generated by neurons, with reported frequencies ranging from 30 to 140 Hz, divided into three distinct gamma bands, including low (30–50 Hz), middle (50–90 Hz) and fast gamma (90–140 Hz)^18^. Increasing evidence indicates that gamma oscillations are prominent in the hippocampus and are essential to memory functions^19,20^. The decreased slow gamma oscillations in the hippocampal CA1 area could weaken memory function^21^. Reduced gamma activity in mouse models and patients with Alzheimer's disease (AD) has also been reported cause memory disorders^22,23^. One promising way to improve patients’ cognitive function might be restoring normal gamma activity. Interestingly, it has been demonstrated that light flicker stimulation at 40 Hz elicits a wider range of gamma neural oscillations, including the frontal lobe and hippocampus, and stronger entrainment than stimulation at other gamma band frequencies^24,25^. In addition, 40 Hz light flicker stimulation can reduce brain inflammation, protect neurons from damage and improve cognitive function^25,26^. Recently, a two-vessel occlusion (2VO) cerebral ischemia model was found to induce a reduction in CA1 slow gamma power^27^, and a continuous reduction in low gamma oscillation was also observed in a mouse model of unilateral hippocampal hypoperfusion^28^. Interestingly, 40 Hz light flicker restored the 2VO-induced decrease in CA1 slow gamma power, protected CA1 neurons from ischemic injury and improved cognitive function^27^.

However, it is still unclear whether 40 Hz light flicker stimulation has a neuroprotective effect on the CCH model and its effect on the hippocampal mRNA expression profile is unknown. Therefore, this study established an experimental mouse model by rUCCAO subjected to stimulation with 40 Hz light flicker for 1 month, and investigated the changes in cognitive function and related mechanisms. The present study may provide a new strategy for the treatment of CCH and may clarify the associated biological mechanisms.

## Methods

### Animals

Male C57BL/6 mice (8 weeks, 20–25 g) were purchased from Hangzhou Hang Si Biotechnology Co., Ltd (Hangzhou, China). Mice were housed in a pathogen-free SPF animal facility in a condition-controlled room (24 ± 1 °C, 50 ± 10% humidity) under a 12 h light–dark cycle (lights on 06:00–18:00). Mice were raised in ventilated cages with 5 mice per cage and given access to food and water.

### Ethics statement

All experimental procedures and animal handling protocols were conducted according to the Animal Research: Reporting In Vivo Experiment: The ARRIVE Guidelines for laboratory animals. These procedures were approved by the Animal Care Committee of The First Affiliated Hospital, Zhejiang University School of Medicine.

### Visual stimulation equipment

The 40 Hz light flicker stimulation equipment was purchased from a Service Center Company (Qingdao, China). By testing the parameters with a photosensitive sensor, we found that the actual flicker frequency was 40 ± 0.1 Hz with a duty cycle of 50% and that the color temperature was within the range of 6000 ± 500 K, satisfying the experimental design requirements. Light flicker stimulation methods were performed as previously described^29^.

### Experimental design and schematic diagram

**Figure 1:**
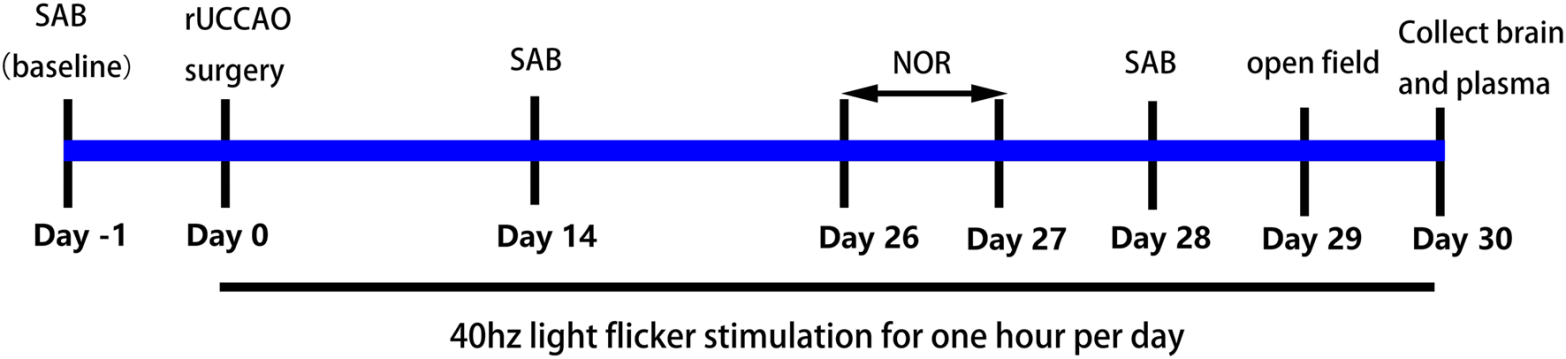
Scheme of the experimental design. *rUCCAO* right unilateral common carotid artery occlusion, *NOR* novel object recognition, *SAB* spontaneous alternation behavior.

A total of 60 mice of the same age (8 weeks old) and sex (male) were randomly divided into two groups (30 mice in each group) and underwent sham surgery or right unilateral common carotid artery occlusion (rUCCAO) surgery. After surgery, the mice in each group were randomly assigned to receive 40 Hz light flicker or normal light treatment. Ultimately, the mice were divided into four groups: sham, sham + 40 Hz, rUCCAO and rUCCAO + 40 Hz. The scheme of the experimental design is depicted in Fig. 1. The first light flicker stimulation was performed 12 h after surgery in the sham + 40 Hz and rUCCAO + 40 Hz groups. The stimulation was conducted once per day between 9–10 a.m. throughout the experimental duration.

### Surgical procedure

Right UCCAO or sham surgery was performed in 8-week-old male mice using aseptic techniques. Following the procedures of a previous report^4^, mice were anesthetized with 1.5% sodium pentobarbital by intraperitoneal injection. After the midline cervical incision, the right common carotid artery was isolated from the veins and vagus nerve, and two 6-0 silk sutures were placed under the carotid. For rUCCAO mice, the two sutures were tightly tied, and the carotid was cut in the middle of the double ligation. For sham mice, the right common carotid artery was only separated. For all mice, incisions were closed, and mice were allowed to recover in a comfortable environment. The mouse survival rate was 100%.

### Behavioral tests

All mice underwent the following behavioral experiments. The arena and objects were cleaned between trials with 75% ethanol to remove olfactory distraction. Some tests were performed under a low-illumination red light to minimize stress levels. A video camera positioned above the arena tracked the mouse’s location. Data were recorded and analyzed using Any-maze software (Stoelting).

#### Novel object recognition (NOR) test

The experimental apparatus was a bare square box (50 cm long, 50 cm wide, 50 cm high). Before starting the experiment, each mouse was placed in the box for 5 min to adapt to the new environment. Then, we placed two similar objects (object A and object B) in the box; each mouse was permitted to explore the two similar objects freely for 5 min. Two hours later, we replaced object B with object C and the mice were permitted a further 5 min to explore the two objects. The recognition index was defined by the percentage of TC/(TA + TC) (TA: time spent exploring object A; TC: time spent exploring object C). Exploration was defined as touching the object with the nose or being within 1 cm of an object. The total distance traveled was recorded to determine whether locomotive deficits disturbed object recognition memory testing, according to a previous report^30^.

#### Spontaneous alternation behavior (SAB) test

Spatial working memory was examined using the spontaneous alternation task in the Y-maze apparatus. The Y-maze is a three-arm maze (40 cm long, 3 cm wide, 12 cm high walls) in which the arms are at a 120-angle from each other. Initially, we placed animals within the center. The number of arm entries and sequences (i.e., BCA) were recorded in a 5-min test phase. A spontaneous alternation was defined as entries into all three arms on consecutive choices (i.e., BCA, ABC, or CAB, but not CAC). The rate of alternations was defined according to the following equation: Alternation rate (%) = [(number of alternations)/(total arm entries-2)] × 100. The number of total arm entries served as an indicator of locomotor activity, according to a previous report^11^.

#### Open field (OF) test

Mouse spontaneous activity in the open field was tested using a bare square box (50 cm long, 50 cm wide, 50 cm high) to judge the emotional reaction of the mice. The center area of the box field was defined as 30 cm × 30 cm. Before starting the experiment, we placed each mouse in the box for 5 min to adapt to the new environment; then, the mouse was given 5 min to move freely in the box. The total distance traveled and the time spent in the inner zone were recorded, according to a previous report^27^.

### Toluidine blue staining

Mice (n = 3/group) were initially perfused intracardially with normal saline and then rapidly perfused with 4% paraformaldehyde. The brain tissues were removed and postfixed with 4% paraformaldehyde at 4 °C for 48 h. The brains were embedded in paraffin, cut into 5 μm thick slices, and stained with toluidine blue. We collected two brain slices from the frontal lobe and hippocampus area per mouse (from every three slices) and used an optical microscope (Olympus, Tokyo, Japan) to accurately count surviving neurons per square millimeter in the right frontal lobe and hippocampus.

### CD31 immunohistochemistry

Mice (n = 3/group) were initially perfused intracardially with normal saline, and then rapidly perfused with 4% paraformaldehyde. The brain tissues were removed and postfixed with 4% paraformaldehyde at 4 °C for 48 h. The brains were embedded in paraffin and cut into 5 μm thick slices. The sections were stained with anti-CD31 antibody (Boster Bioengineering, Wuhan, China) according to the manufacturer's instructions at 4 °C overnight, stained with an appropriate secondary antibody conjugated with horseradish peroxidase and washed with PBS. Next, the sections were stained with diaminobenzidine, counterstained with hematoxylin, dehydrated, subjected to gradient alcohol and xylene dehydration and mounted. We collected two brain slices in the frontal lobe and hippocampus area per mouse (from every three slices) and used an optical microscope (Olympus, Tokyo, Japan) to observe and obtain images. The positive area and integrated optical density (IOD) of CD31 protein in the ischemic hemisphere were analyzed by ImageJ software.

### RNA sequencing of the hippocampus in mice

The hippocampal tissues of mice (n = 3/group) were rapidly removed and weighed, placed in 1.5 mL EP tubes, and frozen at − 80 °C until use. Total RNA was extracted using TRIzol reagent (Invitrogen, CA, USA) according to the manufacturer's protocol. RNA purity and quantification were evaluated using a NanoDrop2000 spectrophotometer (Thermo Scientific, USA). RNA integrity was assessed using an Agilent 2100 Bioanalyzer (Agilent Technologies, Santa Clara, CA, USA). Then, the libraries were constructed using the VAHTS Universal V6 RNA-seq Library Prep Kit according to the manufacturer's instructions. OE Biotech Co., Ltd. (Shanghai, China) conducted the transcriptome sequencing and analysis. A p value < 0.05 and fold change > 2 or fold change < 0.5 were set as the thresholds for significantly differentially expressed genes (DEGs). Database annotation of the functions and pathways of DEGs was carried out by the Gene Ontology (GO)^31^ and Kyoto Encyclopedia of Genes and Genomes (KEGG)^32^ co-enrichment analysis.

### Quantitative real-time PCR (Q-PCR)

Total RNA from hippocampal tissues (n = 6) frozen at − 80 °C was extracted using a FastPure Cell/Tissue Total RNA Isolation Kit V2 (RC112-01, Nanjing, China). A NanoDrop-2000 Spectrophotometer (Thermo Fisher Scientific, USA) was used to determine the total RNA mass (A260/280 ratio: 1.8–2.1, A260/230 ratio > 2.0). Reverse transcription reactions were conducted following the HiScript III RT SuperMix for qPCR with gDNA wiper (R323-01, Nanjing, China) standard procedures. Q-PCR was performed on a CFX384 Q-PCR system (Bio-Rad, Singapore) using ChamQ Universal SYBR qPCR Master Mix (Q711, Nanjing, China). The thermal cycling conditions for Q-PCR were as follows: stage 1: predenaturation at 95 °C for 30 s, stage 2: 40 cycles of 95 °C for 10 s and 60 °C for 30 s, and stage 3: melting curve set according to the conventional instrument settings. Each tissue was analyzed three times to ensure the reliability of the results. We used the mRNA expression of β-actin as an internal control and calculated and analyzed the relative expression of the target gene mRNA by the 2^−ΔΔCT^ method. Primer pairs designed for the Q-PCR analysis are shown in Table 1.

**Table 1:** Primer sequences used for RT-qPCR.

Gene	Primer sequences	Product (bq)	GeneBank no
β-Actin	F:CACTGTCGAGTCGCGTCC R:TCATCCATGGCGAACTGGTG	89	NM_007393.5
IL-1β	F:TGCCACCTTTTGACAGTGATG R:TGATGTGCTGCTGCGAGATT	138	NM_008361.4
TNF-α	F:GATCGGTCCCCAAAGGGATG R:CCACTTGGTGGTTTGTGAGTG	92	NM_001278601.1
Arc	F:GGGTGAGCTGAAGCCACAAA R:ACTGGTATGAATCACTGGGGG	82	NM_001276684.1
Per2	F:CGCGGCGAAGCGCTTATT R:GTGGGACTGGTGGGACTTG	74	NM_001420881.1

### Statistical analysis

Data are expressed as the mean ± standard error of the mean (mean ± SEM). Statistical analyses were conducted using SPSS 20.0 software (SPSS Inc., USA). Statistical significance was determined using one-way analysis of variance (ANOVA) followed by Tukey’s test for multigroup comparisons. *p* < 0.05 indicated a statistically significant difference. Alternation rates in the SAB test were analyzed with repeated measures analysis of variance.

## Results

### 40 Hz light flicker stimulation ameliorated short-term memory impairments in mice with rUCCAO

We examined the effect of the 40 Hz light flicker stimulation on behavioral changes in rUCCAO mice.

**Figure 2:**
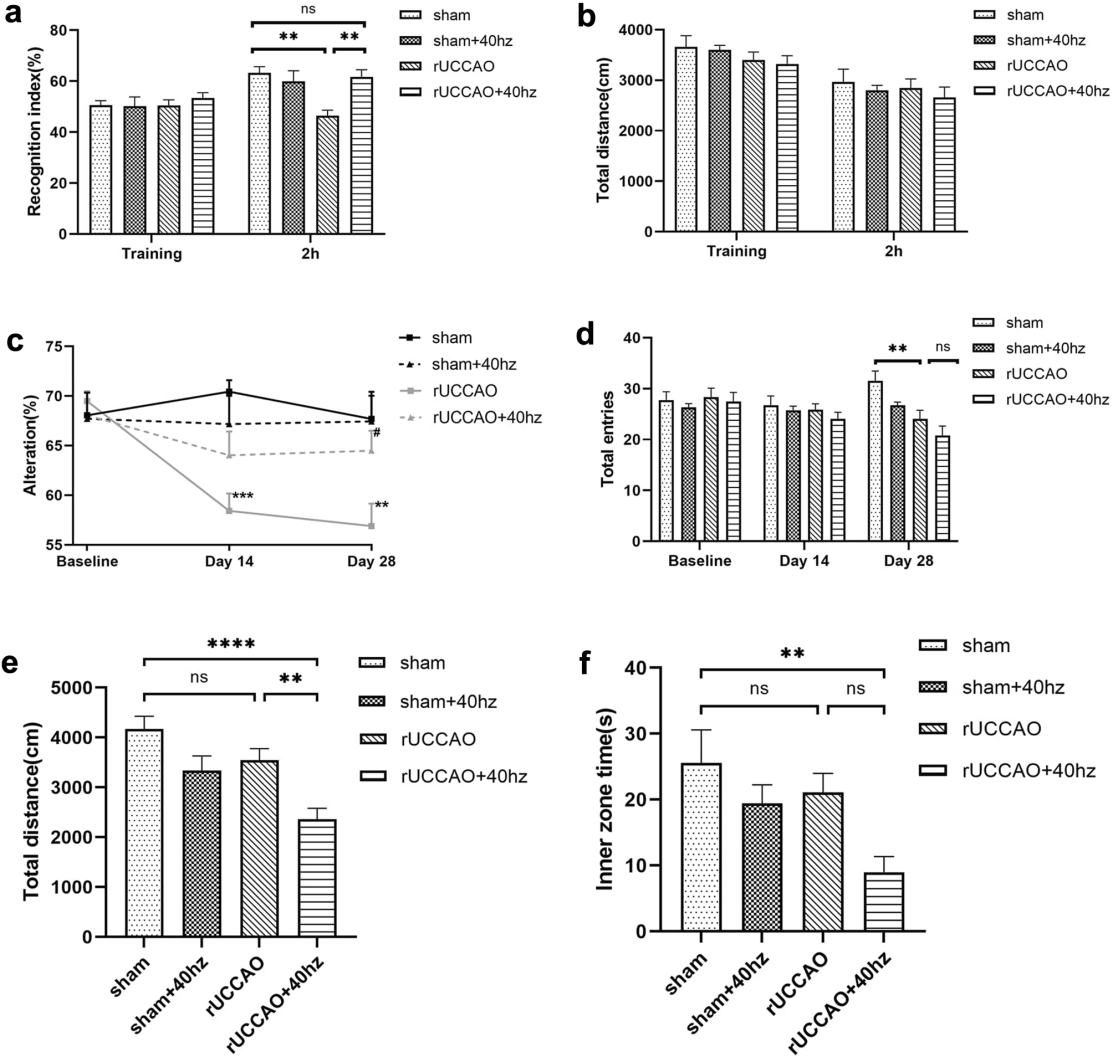
40 Hz light flicker stimulation improved memory impairment in rUCCAO mice. (**a**) Recognition index of NOR in the training and 2 h test stages. (**b**) Running distance in the training and 2 h test stages. (**c**) The alternation rate in the SAB test at baseline and on the 14th and 28th day. (**d**) The total number of entries in the SAB test at baseline and on the 14th and 28th day. (**e**) The total distance traveled in the OF test. (**f**)The time spent in the inner zone in the OF test (Fig. 2c, n = 15/group. Means ± SEMS, **p < 0.01, ***p < 0.001, rUCCAO group vs. sham group; ^#^p < 0.05, rUCCAO + 40 Hz group vs. rUCCAO group. *n.s.* not significant. Figure 2a,b,d–f, n = 15/group. Means ± SEMs, *p < 0.05, **p < 0.01, ***p < 0.001, *n.s*. not significant).

In the NOR test, the experiment was divided into two stages: the training stage and the short-term memory test stage. In the training stage, there were no significant differences in the recognition index among the groups (p > 0.05, Fig. 2a). However, there were significant differences in the recognition index among the four groups in the memory test stage [F (3,56) = 6.366, p < 0.001]. The recognition index was significantly decreased in the rUCCAO group compared to the sham group (p < 0.01, Fig. 2a), and it was increased in the rUCCAO + 40 Hz group compared to the rUCCAO group (p < 0.01, Fig. 2a). There was no significant difference in locomotor activity as determined by the total distance among the four groups (p > 0.05, Fig. 2b), suggesting 40 Hz light flicker stimulation improved short-term memory in rUCCAO + 40 Hz group.

In the spontaneous alternation behavior (SAB) test, we recorded the alternation rate and total entry numbers at baseline and on the 14th and 28th days. As shown in Fig. 2c, we found an interaction between the time and group effects (F = 2.453, p = 0.029). Next, we performed a simple effect analysis on the time and intervention factors. On the one hand, the alternation rate were significantly different at different times (F = 4.144, p < 0.05). Pairwise comparisons showed that the alternation rate was significantly reduced on the 14th day (p < 0.01) and on the 28th day (p < 0.001) compared to the baseline in the rUCCAO group; however, the results in the other groups were not statistically significant. On the other hand, 40 Hz light flicker stimulation had a significant effect on the alternation rate among the four groups [F (3,56) = 5.740, p < 0.01]. Pairwise comparisons showed that the alternation rate of the rUCCAO group was significantly reduced (p < 0.001) compared with that of the sham group, however, there was no significant difference between the rUCCAO and rUCCAO + 40 Hz groups on the 14th day. On the 28th day, the alternation rate of the rUCCAO group was significantly reduced (p < 0.01) compared with that of the sham group; the rate of the rUCCAO + 40 Hz group was significantly increased (p < 0.05) compared with the rUCCAO group. Interestingly, there was a significant difference in locomotor activity on the 28th day among the four groups as determined by total entries [F (3,56) = 7.975, p < 0.01, Fig. 2d]. The results showed that 40 Hz light flicker improved spatial working memory in the rUCCAO + 40 Hz group.

The open field (OF) test showed that there were significant differences in the total distance traveled [F (3, 56) = 8.930, p < 0.001, Fig. 2e] and the time spent in the inner zone [F (3, 56) = 4.186, p < 0.01, Fig. 2f] among the four groups. The rUCCAO + 40 Hz group performed poorly compared with the rUCCAO group in terms of the total distance traveled (p < 0.01, Fig. 2e) or time spent in the inner zone (p = 0.0716, Fig. 2f). However, there were no significant differences in the total distance travelled and the time spent in the inner zone between the rUCCAO and sham groups (both p > 0.05), demonstrating that 40 Hz light flicker treatment impaired OF performance and produced anxiety-like behaviors in the rUCCAO + 40 Hz group.

### 40 Hz light flicker stimulation rescued frontal lobe and hippocampal pyramidal neuron loss in mice with rUCCAO

**Figure 3:**
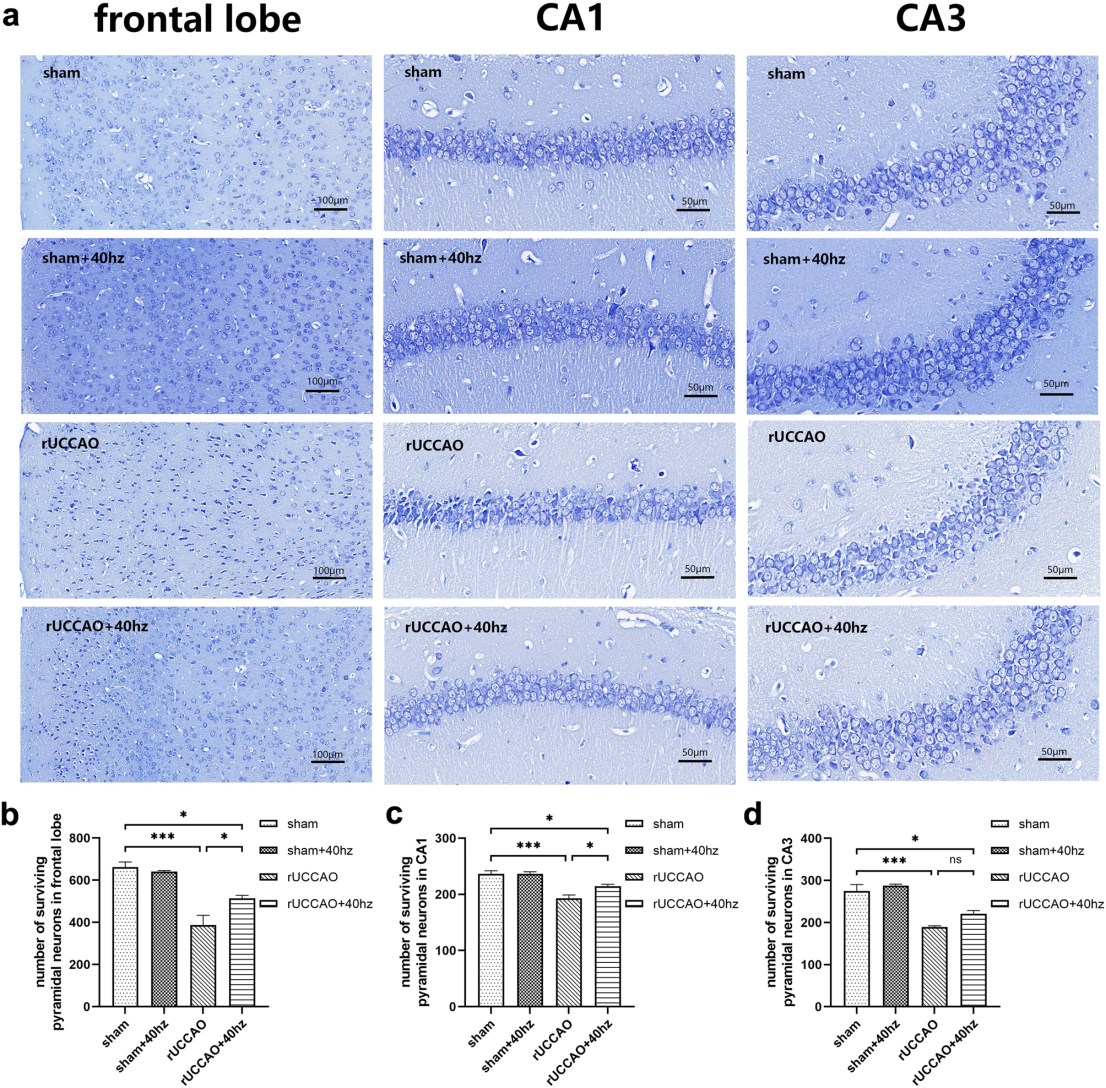
40 Hz light flicker stimulation alleviated the damage to neurons in the frontal lobe and hippocampus in mice with rUCCAO. (**a**) Toluidine blue staining in the frontal lobe, and CA1 and CA3 regions, scale bar = 50 μm or 100 μm. (**b**–**d**) The number of surviving pyramidal neurons in the frontal lobe, CA1 and CA3 regions (n = 3/group. Means ± SEMs, *p < 0.05, ***p < 0.001, *n.s*. not significant).

We next used toluidine blue staining to investigate histomorphological changes to confirm whether 40 Hz light flicker stimulation alleviated the damage to the frontal lobe and hippocampal pyramidal neurons. In the sham group, the pyramidal neurons were stained deep blue and tightly arranged with normal cellular microstructure in the frontal lobe, CA1 and CA3 regions. In the rUCCAO group, the surviving pyramidal neurons were stained light blue and loosely arranged with no definite shape (Fig. 3a). There were significant differences in the number of surviving neurons among the groups (frontal lobe: F (3, 8) = 21.44, p < 0.001; CA1: F (3, 8) = 19.65, p < 0.001; CA3: F (3, 8) = 27.23, p < 0.001, Fig. 3b–d). The number of surviving neurons in the frontal lobe, CA1 and CA3 regions was significantly decreased in the rUCCAO group compared to the sham group (all p < 0.001). Interestingly, 40 Hz light flicker treatment restored morphological damage and significantly attenuated pyramidal neuron loss (frontal lobe: p < 0.05; CA1: p < 0.05; CA3: p > 0.05) in the rUCCAO + 40 Hz group; however, the number of normal neurons in the rUCCAO + 40 Hz group was still lower than that in the sham group (all p < 0.05). These data indicated that 40 Hz light flicker stimulation could alleviate the damage to neurons in the frontal lobe, CA1 and CA3 regions after rUCCAO.

### 40 Hz light flicker stimulation does not induce angiogenesis in mice with rUCCAO

**Figure 4:**
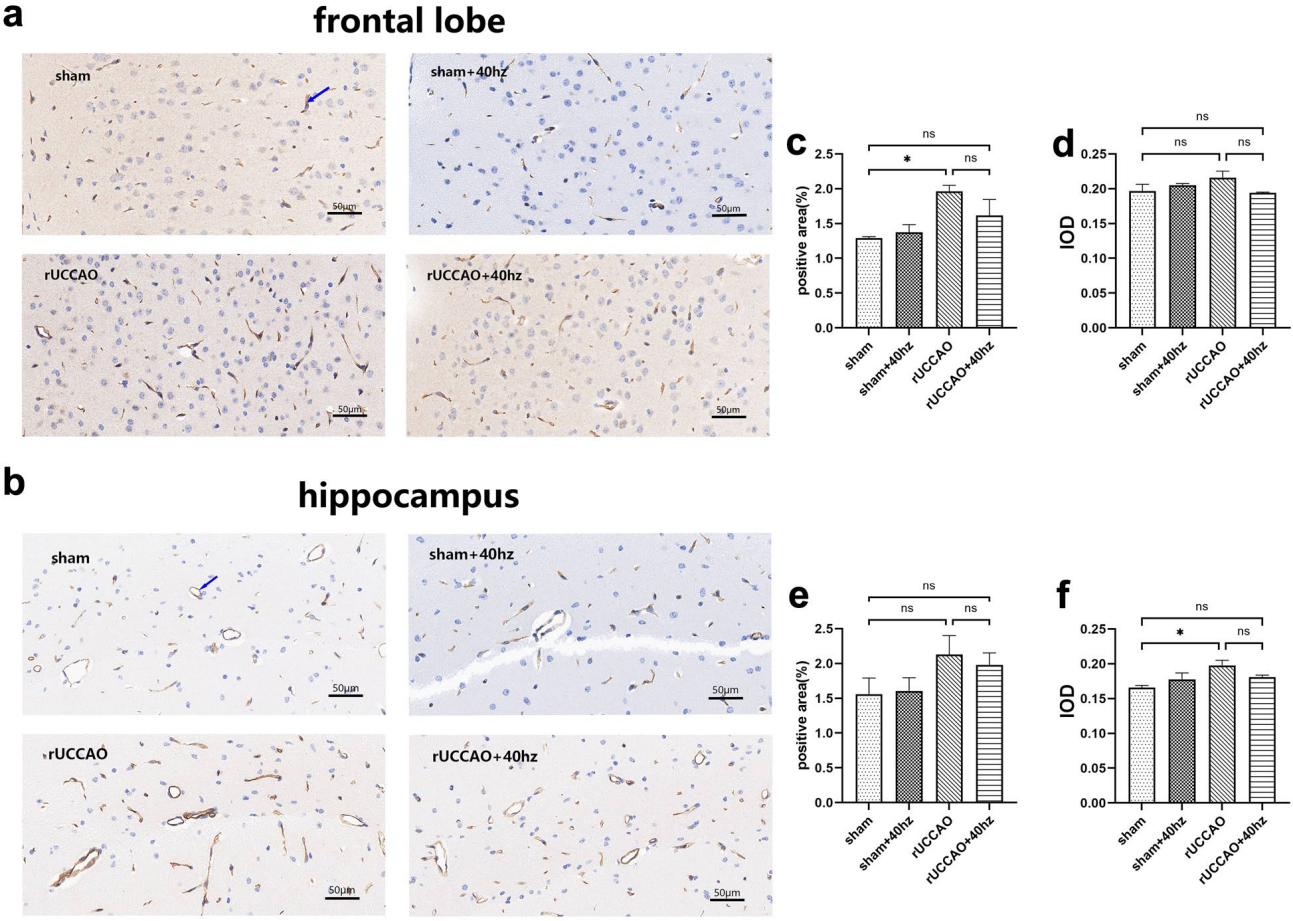
Light flicker stimulation at 40 Hz does not increase angiogenesis in mice with rUCCAO. (**a**,**b**) CD31 immunohistochemical staining in the frontal lobe and hippocampus. Scale bar = 50 μm; the blue arrow indicates the positive area. (**c**–**f**) Positive area (%) and IOD of CD31 protein in the frontal lobe and hippocampus, respectively (n = 3/group. Means ± SEMs, *p < 0.05, *n.s.* not significant).

To determine the effect of 40 Hz light flicker stimulation on angiogenesis, we next investigated the CD31 protein level in the frontal lobe and hippocampus by immunohistochemistry (Fig. 4a,b). The positive area in the frontal lobe was significantly different among the groups [F (3, 8) = 4.951, p < 0.05, Fig. 4c]. The positive area in the rUCCAO group was significantly increased compared with that in the sham group (p < 0.05, Fig. 4c); however, compared with the rUCCAO group, 40 Hz light flicker stimulation had no significant effect on the CD31 protein-positive area (p > 0.05, Fig. 4c). The integrated optical density (IOD) in the hippocampus was significantly different among the groups [F (3,8) = 4.470, p < 0.05, Fig. 4f]. The IOD of the rUCCAO group was significantly lower than that of the sham group (p < 0.05, Fig. 4f). However, there were no significant differences in IOD between the rUCCAO and rUCCAO + 40 Hz groups (p > 0.05, Fig. 4f). These data suggest that 40 Hz light flicker stimulation does not induce angiogenesis in mice with rUCCAO.

### DEG analysis in the hippocampus

Total RNA was extracted from the hippocampus and transcriptome sequencing was performed. In total, 62.57 Gb clean data were obtained, with 6.89–7.01 Gb per sample. In sequence alignment analysis, the ratio of clean reads that were mapped to the reference genome was 97.52–98.61%, the Q30 ranged from 93.85 to 94.49% and the average GC content was 49.14%, indicating that the RNA-sequencing results were reliable.

**Figure 5:**
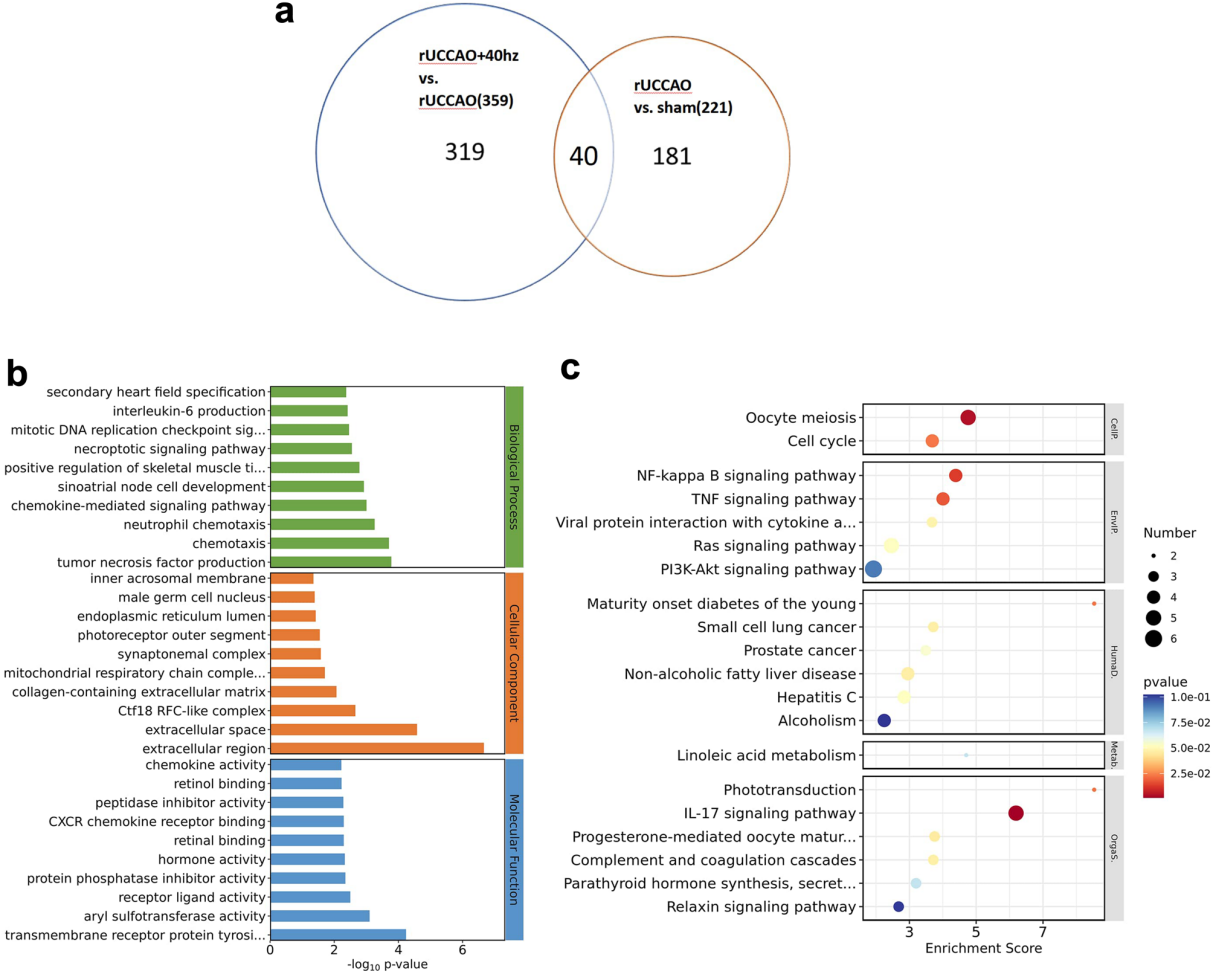
Effect of 40 Hz light flicker stimulation on the hippocampal transcriptome in rUCCAO mice. (**a**) The Venn diagram shows the number of identified DEGs. (**b**) Top 30 GO terms, rUCCAO + 40 Hz vs. rUCCAO group. The Y axis on the left indicates the GO terms, the Y-axis on the right indicates the three GO categories, and the X-axis indicates the p value. (**c**) Top 20 KEGG enriched pathways scatter plot of DEGs, rUCCAO + 40 Hz vs. rUCCAO group. Each circle in the figure represents a KEGG pathway. The X-axis indicates the enrichment score, the Y-axis on the left represents the name of the pathway and the X-axis on the right indicates KEGG categories.

When compared to the rUCCAO group, 221 genes were differentially expressed (138 upregulated and 83 downregulated) in the sham group and 359 genes (60 upregulated and 299 downregulated) in the rUCCAO + 40 Hz group, with a fold change > 2 and p < 0.05 (Fig. 5a). Interestingly, we found that 40 Hz light flicker stimulation induced cognition-related gene changes in the rUCCAO mice, as shown in Table 2, including Arc and Per2 genes, when we investigated the function of DEGs with fold change > 1.5 and p < 0.05.

**Table 2:** Differentially expressed genes related to cognitive function in rUCCAO + 40 Hz group compared to rUCCAO group.

Gene ID	Gene symbol	Regulation	log2FoldChange	p-value	q-value
11838	Arc	Up	0.65	1.90E−05	0.042
18627	Per2	Up	0.93	1.97E−11	3.96E−07

### GO and KEGG functional and pathway enrichment analyses

GO and KEGG enrichment analyses were conducted to explore the biological functions of the DEGs. The results of GO analysis and KEGG enrichment analysis can be found in Supplementary GO enrichment, KEGG enrichment and annotation.

In the GO analysis between the rUCCAO and rUCCAO + 40 Hz groups, 188 DEGs were classified into three GO categories and 1325 terms, and the 10 most significant terms from each category are summarized in Fig. 5b. The most significant GO biological process (BP) term in which the genes were enriched was tumor necrosis factor production (GO:0032640). The most significant cellular component (CC) term was extracellular region (GO:0005576). The most significant molecular function (MF) term was transmembrane receptor protein tyrosine kinase activator activity (GO:0030297). Similarly, in the KEGG enrichment analysis between the rUCCAO + 40 Hz and rUCCAO groups, 76 DEGs were annotated to 183 pathways in the KEGG pathway database, and the top 20 enriched pathways are shown in Fig. 5c. The top 4 enriched pathways were oocyte meiosis (mmu04114), NF-kappa B signaling pathway (mmu04064), IL-17 signaling pathway (mmu04657) and TNF signaling pathway (mmu04668), suggesting that 40 Hz light flicker has a substantial impact on the inflammation-related pathway in mice with rUCCAO.

### Verification of selected genes and mRNA expression related to neuroinflammation

**Figure 6:**
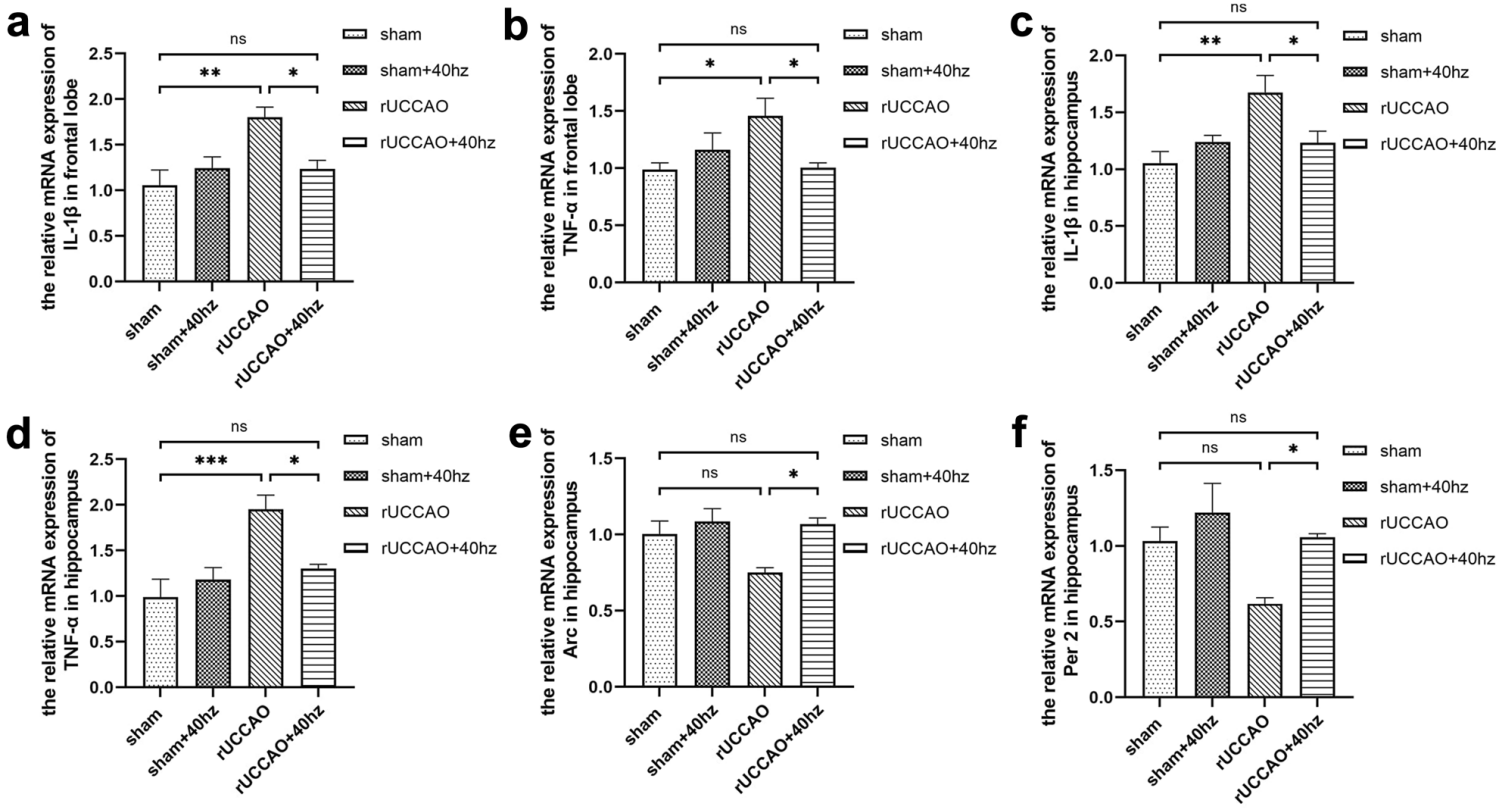
Verification of candidate DEGs and neuroinflammation-related genes using Q-PCR. (**a**–**d**) Relative IL-1β and TNF-α mRNA expression in the frontal lobe and hippocampus. (**e**,**f**) Relative Arc and Per2 mRNA expression in the hippocampus (n = 6/group, Means ± SEMs, *p < 0.05, **p < 0.01, ***p < 0.001, *n.s*. not significant).

Q-PCR was executed to show the reliability of the RNA-seq results. A total of two DEGs related to cognitive function (Table 2) were identified. The relative mRNA expression of the genes confirmed through Q-PCR experiments was consistent with the RNA sequencing results (Fig. 6e,f). This suggests that our transcriptome sequencing results have reference significance. The relative mRNA expression of TNF-α and IL-1β in the hippocampus and frontal lobe was markedly increased (p < 0.05, Fig. 6a–d) in the rUCCAO group compared to the sham group. However, administration of the 40 Hz light flicker decreased (p < 0.05, Fig. 6a–d) the expression of TNF-α and IL-1β in the hippocampus and frontal lobe. These data suggested that 40 Hz light flicker can reduce the secretion of inflammatory cytokines and improve neuroinflammation in rUCCAO mice.

## Discussion

The current study aimed to elucidate the neuroprotective effect and mechanism of 40 Hz light flicker on VCI using a mouse model of CCH induced by rUCCAO. First, we observed that 40 Hz light flicker stimulation could improve short-term and spatial working memory in mice with rUCCAO and protect neurons in the frontal lobe and hippocampus from damage. Second, immunohistochemical staining showed that its neuroprotective effect was not related to angiogenesis. Finally, hippocampal transcriptome sequencing and Q-PCR results showed that 40 Hz light flicker ameliorated CCH-induced neuroinflammation and affected the expression of cognition-related genes.

The rUCCAO mouse model used in this study induces a sustained decrease in ipsilateral cerebral blood flow, leading to cognitive impairment, and has been demonstrated to be an ideal model for investigating the effects of long-term treatment on VCI^10,33^. In addition, the advantage of the rUCCAO model is that damage to the endothelium is avoided and the survival rate is 100%^9^. Successful occlusion was assessed by a maker of ipsilateral eyelid drooping that started immediately after successful occlusion and was sustained over 20 weeks, as previously reported^34^.

Our study showed that rUCCAO mice exhibited short-term and spatial working memory impairment. This result is consistent with those of other studies in which CCH induced by rUCCAO was associated with cognitive deficits^8,11^. Previous studies have shown that 40 Hz light flicker stimulation can improve cognitive function in mice with CK-p25 and Tau P301S neurodegenerative diseases^25^, transgenic 3×Tg-AD mice^26^ and stroke mice^27^. We found that 40 Hz light flicker stimulation improved short-term memory in the NOR test and spatial working memory in the SAB test in mice with rUCCAO, indicating that 40 Hz light flicker stimulation positively ameliorates memory impairment caused by CCH. However, the OF results showed that 40 Hz light flicker stimulation caused anxiety in rUCCAO mice. It has been reported^35^ that 40 Hz light flicker stimulation acts as a stressor and could activate stress-related brain regions in the hippocampus and hypothalamus and increase blood cortisol levels. We suggest that it is necessary to optimize light stimulation equipment and explore optimal stimulation parameters to reduce the emotional changes in future research.

The frontal lobe and hippocampus are closely related to cognitive function^36,37^. Cortical and hippocampal neurons are particularly sensitive to cerebral ischemia^38^. Recent studies have shown that neurons and white matter exhibit loose arrangement and loss after 1 month of rUCCAO, and learning and memory impairment^30^, which is consistent with our research findings. Interestingly, 40 Hz light flicker stimulation restored the normal morphology of neurons and reduced neuronal loss in the frontal lobe and hippocampus in mice with rUCCAO. Zheng et al. also reported that 40 Hz light flicker stimulation can protect hippocampal CA1 neurons in stroke mice^27^. Our results demonstrated that 40 Hz light flicker stimulation had a protective effect on neurons in CCH mice. However, whether 40 Hz light flicker stimulation has a protective effect on white matter and its effects on glial cells in CCH models need to be determined in future studies.

According to a previous report, postischemic angiogenesis is involved in the axonal outgrowth and the proliferation, migration, and maturation of neural stem cells and is correlated with longer survival times in ischemic stroke patients^39^. CD31 is mainly used to demonstrate the presence of endothelial cell tissue in immunohistochemistry and evaluate tumor angiogenesis^40^. However, we did not observe angiogenesis in rUCCAO + 40 Hz group mice when performing CD31 immunohistochemical staining, which suggest 40 Hz light flicker stimulation is not beneficial to the underlying vascular issues. Immunostaining of a marker of the vascular endothelium, lectin, revealed increased blood vessel diameters in the auditory cortex and CA1 after 40 Hz chronic auditory stimulation-induced gamma entrainment in 5XFAD mice^41^. Therefore, it is meaningful to study the effect of 40 Hz light flicker stimulation on blood vessels in CCH models.

The results of the hippocampal transcriptome analysis by RNA sequencing showed that there were 221 DEGs between the sham and rUCCAO groups and 359 DEGs between the rUCCAO and rUCCAO + 40 Hz groups. GO annotation data revealed changes in the expression of genes related to tumor necrosis factor production (GO:0032640), transmembrane receptor protein tyrosine kinase activator activity (GO:0030297) and extracellular region (GO:0005576) between the rUCCAO and rUCCAO + 40 Hz groups. KEGG pathway analysis identified oocyte meiosis (mmu04114), the NF-kappa B signaling pathway (mmu04064), the IL-17 signaling pathway (mmu04657) and the TNF signaling pathway (mmu04668) as notable pathways. Therefore, 40 Hz light flicker stimulation may play an important role in the regulation of inflammation-related pathways in mice with rUCCAO. In addition, 40 Hz light flicker stimulation induced cognition-related genes, including Arc and Per2. The Arc gene (also known as-Arg3.1) is a main player in both synaptic plasticity processes that can sustain learning and memory-related cognitive processes^42^; Per2 KO mice exhibited impaired spatial working memory, indicating that Per2 expression levels might influence spatial working memory performance^43^, confirming that the 40 Hz light flicker is beneficial to cognitive function in CCH mice.

Neuroinflammation is characterized by a series of proinflammatory cytokines and chemokines produced by intrinsic brain cells such as microglia and astrocytes, as well as the infiltration of peripheral immune cells into the central nervous system^44^. Previous studies have found that 40 Hz light flicker stimulation can reduce neuroinflammation, achieving neuroprotection in neurodegenerative mice^25^ and transgenic AD mice^26^; in addition, 40 Hz light flicker stimulation can regulate cytokine expression profiles, including TNF-α and IL-1β^45^. Our Q-PCR results showed that 40 Hz light flicker stimulation reduced the expression of the inflammatory mediators TNF-α and IL-1β in the frontal lobe and hippocampus, indicating that 40 Hz light flicker stimulation can improve neuroinflammation in mice with rUCCAO. However, our results may not fully reflect complex inflammatory changes because they rely on two neuroinflammatory mediators, although TNF-α and IL-1β play an important role in propagating neuroinflammation^44^. Therefore, there is still a need for a further detailed study of this topic.

The limitations of this study are as follows: First, we may not be able to capture the entire human condition using a single sex and strain of mice. Second, our cognitive assessment is limited to memory and does not address other cognitive domains such as learning. Third, hippocampal atrophy and ventricular dilation occurred 4 months after rUCCAO^10^; whether 40 Hz light flicker treatment can still play a neuroprotective role in the long-term and the optimal stimulation parameters of the treatment are unknown. In addition, we did not conduct electrophysiological experiments to test the direct effect of 40 Hz light flicker stimulation on gamma entrainment in rUCCAO mice, which depended on previous research data. Finally, the mechanism of memory amelioration remains insufficiently explored due to the sample size, and it is necessary to study the related mechanism in the future.

## Conclusion

In conclusion, 40 Hz light flicker is able to suppress short-term and spatial working memory impairment and protect neurons from ischemic damage due to chronic cerebral hypoperfusion. This effect may be associated with the attenuation of neuroinflammation and the regulation of cognitive-related gene expression.

### Supplementary Information

Supplementary Information 1.

Supplementary Information 2.

## Data Availability

The datasets used and/or analyzed during the current study are available from the corresponding author upon reasonable request.
