# Supplementary material for: Effect of 40 Hz light flicker on cognitive impairment and transcriptome of hippocampus in right unilateral common carotid artery occlusion mice
Source: Sci Rep. 2023 Dec 4;13:21361. doi: 10.1038/s41598-023-48897-6 (PMC10695931; doi:10.1038/s41598-023-48897-6)
Supplement: Supplementary file 1 — Supplementary Information 1. [file 41598_2023_48897_MOESM1_ESM.pdf]

Supplementary GO enrichment and annotation

ID	Term	Category	p-value
GO:0005576	extracellular region	cellular component	2.17E-07
GO:0005615	extracellular space	cellular component	2.62E-05
GO:0030297	transmembrane receptor protein tyrosine kinase activator activity	molecular function	5.83E-05
GO:0032640	tumor necrosis factor production	biological process	0.000166647
GO:0006935	chemotaxis	biological process	0.000198083
GO:0030593	neutrophil chemotaxis	biological process	0.000550817
GO:0004062	aryl sulfotransferase activity	molecular function	0.000794781
GO:0070098	chemokine-mediated signaling pathway	biological process	0.000988932
GO:0060931	sinoatrial node cell development	biological process	0.001185095
GO:0048633	positive regulation of skeletal muscle tissue growth	biological process	0.00164929
GO:0031390	Ctf18 RFC-like complex	cellular component	0.002186018
GO:0097527	necroptotic signaling pathway	biological process	0.002793945
GO:0048018	receptor ligand activity	molecular function	0.003154858
GO:0033314	mitotic DNA replication checkpoint signaling	biological process	0.003471758
GO:0032635	interleukin-6 production	biological process	0.003821657
GO:0003139	secondary heart field specification	biological process	0.004218161
GO:0032872	regulation of stress-activated MAPK cascade	biological process	0.004218161
GO:0060379	cardiac muscle cell myoblast differentiation	biological process	0.004218161
GO:0004864	protein phosphatase inhibitor activity	molecular function	0.004477554
GO:0005179	hormone activity	molecular function	0.004675253
GO:0031333	negative regulation of protein-containing complex assembly	biological process	0.004829444
GO:0016918	retinal binding	molecular function	0.005031873
GO:0019370	leukotriene biosynthetic process	biological process	0.005031873
GO:0032725	positive regulation of granulocyte macrophage colony-stimulating factor production	biological process	0.005031873
GO:0045236	CXCR chemokine receptor binding	molecular function	0.005031873
GO:0097398	cellular response to interleukin-17	biological process	0.005031873
GO:0030414	peptidase inhibitor activity	molecular function	0.005187679
GO:0010466	negative regulation of peptidase activity	biological process	0.005738674
GO:0019841	retinol binding	molecular function	0.005911634
GO:0008009	chemokine activity	molecular function	0.005983395
GO:0003215	cardiac right ventricle morphogenesis	biological process	0.006856199
GO:0003688	DNA replication origin binding	molecular function	0.007864339
GO:0033147	negative regulation of intracellular estrogen receptor signaling pathway	biological process	0.007864339
GO:0007599	hemostasis	biological process	0.008245258
GO:0062023	collagen-containing extracellular matrix	cellular component	0.008566795
GO:0030234	enzyme regulator activity	molecular function	0.008749871
GO:0002456	T cell mediated immunity	biological process	0.008934845
GO:0005159	insulin-like growth factor receptor binding	molecular function	0.008934845
GO:0007188	adenylate cyclase-modulating G protein-coupled receptor signaling pathway	biological process	0.009272223
GO:0001523	retinoid metabolic process	biological process	0.010066521
GO:0008324	monoatomic cation transmembrane transporter activity	molecular function	0.010066521

GO:0001755	neural crest cell migration	biological process	0.010946907
GO:0045725	positive regulation of glycogen biosynthetic process	biological process	0.011258189
GO:0051146	striated muscle cell differentiation	biological process	0.011258189
GO:0007601	visual perception	biological process	0.012022745
GO:0006123	mitochondrial electron transport, cytochrome c to oxygen	biological process	0.012508686
GO:0097284	hepatocyte apoptotic process	biological process	0.012508686
GO:0008146	sulfotransferase activity	molecular function	0.01343476
GO:0097191	extrinsic apoptotic signaling pathway	biological process	0.01343476
GO:0036122	BMP binding	molecular function	0.013816867
GO:0048557	embryonic digestive tract morphogenesis	biological process	0.013816867
GO:0050830	defense response to Gram-positive bacterium	biological process	0.014033397
GO:0051607	defense response to virus	biological process	0.014078979
GO:0010043	response to zinc ion	biological process	0.0151816
GO:0030111	regulation of Wnt signaling pathway	biological process	0.0151816
GO:0042981	regulation of apoptotic process	biological process	0.015588582
GO:0050896	response to stimulus	biological process	0.016546331
GO:0060384	innervation	biological process	0.016601771
GO:2000406	positive regulation of T cell migration	biological process	0.016601771
GO:0032755	positive regulation of interleukin-6 production	biological process	0.017121285
GO:0007602	phototransduction	biological process	0.018076279
GO:0035066	positive regulation of histone acetylation	biological process	0.018076279
GO:0048020	CCR chemokine receptor binding	molecular function	0.018076279
GO:0051321	meiotic cell cycle	biological process	0.01828395
GO:0004175	endopeptidase activity	molecular function	0.018724096
GO:0032760	positive regulation of tumor necrosis factor production	biological process	0.019278333
GO:0005751	mitochondrial respiratory chain complex IV	cellular component	0.01960404
GO:0006952	defense response	biological process	0.020417039
GO:0071222	cellular response to lipopolysaccharide	biological process	0.020925564
GO:0051923	sulfation	biological process	0.021183984
GO:0006954	inflammatory response	biological process	0.022309182
GO:0004602	glutathione peroxidase activity	molecular function	0.022815056
GO:0007519	skeletal muscle tissue development	biological process	0.023580744
GO:0046628	positive regulation of insulin receptor signaling pathway	biological process	0.024496216
GO:0007015	actin filament organization	biological process	0.025381034
GO:0005158	insulin receptor binding	molecular function	0.026226438
GO:0016064	immunoglobulin mediated immune response	biological process	0.026226438
GO:1900017	positive regulation of cytokine production involved in inflammatory response	biological process	0.026226438
GO:0000795	synaptonemal complex	cellular component	0.026374375
GO:0032024	positive regulation of insulin secretion	biological process	0.027343275
GO:0045087	innate immune response	biological process	0.027483365
GO:0060907	positive regulation of macrophage cytokine production	biological process	0.02800471
GO:0001750	photoreceptor outer segment	cellular component	0.028330983
GO:0006953	acute-phase response	biological process	0.029830035
GO:0016597	amino acid binding	molecular function	0.029830035
GO:0045216	cell-cell junction organization	biological process	0.029830035
GO:0048870	cell motility	biological process	0.029830035
GO:0007596	blood coagulation	biological process	0.031406636
GO:0031016	pancreas development	biological process	0.031701428
GO:0090090	negative regulation of canonical Wnt signaling pathway	biological process	0.031764271
GO:0000976	transcription cis-regulatory region binding	molecular function	0.032871979

GO:0043425	bHLH transcription factor binding	molecular function	0.033617921
GO:0045840	positive regulation of mitotic nuclear division	biological process	0.033617921
GO:0006479	protein methylation	biological process	0.037582391
GO:0005788	endoplasmic reticulum lumen	cellular component	0.038060451
GO:0032147	activation of protein kinase activity	biological process	0.039628496
GO:0035025	positive regulation of Rho protein signal transduction	biological process	0.039628496
GO:0048754	branching morphogenesis of an epithelial tube	biological process	0.039628496
GO:0001673	male germ cell nucleus	cellular component	0.04042567
GO:0043433	negative regulation of DNA-binding transcription factor activity	biological process	0.041635598
GO:0004540	ribonuclease activity	molecular function	0.041715954
GO:0001530	lipopolysaccharide binding	molecular function	0.043843863
GO:0042923	neuropeptide binding	molecular function	0.043843863
GO:0001842	neural fold formation	biological process	0.044292809
GO:0002079	inner acrosomal membrane	cellular component	0.044292809
GO:0002361	CD4-positive, CD25-positive, alpha-beta regulatory T cell differentiation	biological process	0.044292809
GO:0002793	positive regulation of peptide secretion	biological process	0.044292809
GO:0004464	leukotriene-C4 synthase activity	molecular function	0.044292809
GO:0004977	melanocortin receptor activity	molecular function	0.044292809
GO:0005163	nerve growth factor receptor binding	molecular function	0.044292809
GO:0006663	platelet activating factor biosynthetic process	biological process	0.044292809
GO:0008065	establishment of blood-nerve barrier	biological process	0.044292809
GO:0008228	opsonization	biological process	0.044292809
GO:0010749	regulation of nitric oxide mediated signal transduction	biological process	0.044292809
GO:0010750	positive regulation of nitric oxide mediated signal transduction	biological process	0.044292809
GO:0010961	intracellular magnesium ion homeostasis	biological process	0.044292809
GO:0018119	peptidyl-cysteine S-nitrosylation	biological process	0.044292809
GO:0031056	regulation of histone modification	biological process	0.044292809
GO:0031265	CD95 death-inducing signaling complex	cellular component	0.044292809
GO:0032634	interleukin-5 production	biological process	0.044292809
GO:0032831	positive regulation of CD4-positive, CD25-positive, alpha-beta regulatory T cell differentiation	biological process	0.044292809
GO:0032914	positive regulation of transforming growth factor beta1 production	biological process	0.044292809
GO:0035662	Toll-like receptor 4 binding	molecular function	0.044292809
GO:0038060	nitric oxide-cGMP-mediated signaling pathway	biological process	0.044292809
GO:0043152	induction of bacterial agglutination	biological process	0.044292809
GO:0043273	CTPase activity	molecular function	0.044292809
GO:0046633	alpha-beta T cell proliferation	biological process	0.044292809
GO:0048936	peripheral nervous system neuron axonogenesis	biological process	0.044292809
GO:0051347	positive regulation of transferase activity	biological process	0.044292809
GO:0051601	exocyst localization	biological process	0.044292809
GO:0060267	positive regulation of respiratory burst	biological process	0.044292809
GO:0060478	acrosomal vesicle exocytosis	biological process	0.044292809
GO:0060537	muscle tissue development	biological process	0.044292809
GO:0060676	ureteric bud formation	biological process	0.044292809
GO:0060964	regulation of miRNA-mediated gene silencing	biological process	0.044292809
GO:0061088	regulation of sequestering of zinc ion	biological process	0.044292809
GO:0065004	protein-DNA complex assembly	biological process	0.044292809
GO:0071547	piP-body	cellular component	0.044292809

GO:0071548	response to dexamethasone	biological process	0.044292809
GO:0071723	lipopeptide binding	molecular function	0.044292809
GO:0072164	mesonephric tubule development	biological process	0.044292809
GO:1901877	negative regulation of calcium ion binding	biological process	0.044292809
GO:1990111	spermatoproteasome complex	cellular component	0.044292809
GO:2000467	positive regulation of glycogen (starch) synthase activity	biological process	0.044292809
GO:0007129	homologous chromosome pairing at meiosis	biological process	0.046011331
GO:2001244	positive regulation of intrinsic apoptotic signaling pathway	biological process	0.046011331
GO:0051482	positive regulation of cytosolic calcium ion concentration involved in phospholipase C-activating G protein-coupled signaling pathway	biological process	0.048217479
GO:0002376	immune system process	biological process	0.049558882
GO:0033209	tumor necrosis factor-mediated signaling pathway	biological process	0.050461443
GO:0043066	negative regulation of apoptotic process	biological process	0.051025023
GO:0002027	regulation of heart rate	biological process	0.052742369
GO:0045214	sarcomere organization	biological process	0.052742369
GO:0000808	origin recognition complex	cellular component	0.052914352
GO:0001714	endodermal cell fate specification	biological process	0.052914352
GO:0002081	outer acrosomal membrane	cellular component	0.052914352
GO:0002669	positive regulation of T cell anergy	biological process	0.052914352
GO:0003337	mesenchymal to epithelial transition involved in metanephros morphogenesis	biological process	0.052914352
GO:0004030	aldehyde dehydrogenase [NAD(P)+] activity	molecular function	0.052914352
GO:0005166	neurotrophin p75 receptor binding	molecular function	0.052914352
GO:0005732	sno(s)RNA-containing ribonucleoprotein complex	cellular component	0.052914352
GO:0007199	G protein-coupled receptor signaling pathway coupled to cGMP nucleotide second messenger	biological process	0.052914352
GO:0009620	response to fungus	biological process	0.052914352
GO:0009822	alkaloid catabolic process	biological process	0.052914352
GO:0010792	DNA double-strand break processing involved in repair via single-strand annealing	biological process	0.052914352
GO:0014894	onse to denervation involved in regulation of muscle adapta	biological process	0.052914352
GO:0015129	lactate transmembrane transporter activity	molecular function	0.052914352
GO:0016338	calcium-independent cell-cell adhesion via plasma membrane cell-adhesion molecules	biological process	0.052914352
GO:0016936	galactoside binding	molecular function	0.052914352
GO:0019212	phosphatase inhibitor activity	molecular function	0.052914352
GO:0021559	trigeminal nerve development	biological process	0.052914352
GO:0030893	meiotic cohesin complex	cellular component	0.052914352
GO:0031133	regulation of axon diameter	biological process	0.052914352
GO:0031581	hemidesmosome assembly	biological process	0.052914352
GO:0032649	regulation of type II interferon production	biological process	0.052914352
GO:0032782	bile acid secretion	biological process	0.052914352
GO:0032792	negative regulation of CREB transcription factor activity	biological process	0.052914352
GO:0035003	subapical complex	cellular component	0.052914352
GO:0035965	cardiolipin acyl-chain remodeling	biological process	0.052914352
GO:0043256	laminin complex	cellular component	0.052914352
GO:0047198	cysteine-S-conjugate N-acetyltransferase activity	molecular function	0.052914352
GO:0048263	determination of dorsal identity	biological process	0.052914352
GO:0048793	pronephros development	biological process	0.052914352

GO:0051293	establishment of spindle localization	biological process	0.052914352
GO:0051525	NFAT protein binding	molecular function	0.052914352
GO:0060266	negative regulation of respiratory burst involved in inflammatory response	biological process	0.052914352
GO:0060390	regulation of SMAD protein signal transduction	biological process	0.052914352
GO:0060545	positive regulation of necroptotic process	biological process	0.052914352
GO:0060706	cell differentiation involved in embryonic placenta development	biological process	0.052914352
GO:0070296	sarcoplasmic reticulum calcium ion transport	biological process	0.052914352
GO:0090027	negative regulation of monocyte chemotaxis	biological process	0.052914352
GO:0090031	positive regulation of steroid hormone biosynthetic process	biological process	0.052914352
GO:1900086	positive regulation of peptidyl-tyrosine autophosphorylation	biological process	0.052914352
GO:1901895	negative regulation of ATPase-coupled calcium transmembrane transporter activity	biological process	0.052914352
GO:1902751	positive regulation of cell cycle G2/M phase transition	biological process	0.052914352
GO:0005125	cytokine activity	molecular function	0.053513728
GO:0051092	ositive regulation of NF-kappaB transcription factor activit	biological process	0.055062029
GO:0005575	cellular component	cellular component	0.055738301
GO:0070374	positive regulation of ERK1 and ERK2 cascade	biological process	0.061242172
GO:0000076	DNA replication checkpoint signaling	biological process	0.061458529
GO:0001848	complement binding	molecular function	0.061458529
GO:0002725	negative regulation of T cell cytokine production	biological process	0.061458529
GO:0003402	planar cell polarity pathway involved in axis elongation	biological process	0.061458529
GO:0005501	retinoid binding	molecular function	0.061458529
GO:0005746	mitochondrial respirasome	cellular component	0.061458529
GO:0008467	[heparan sulfate]-glucosamine 3-sulfotransferase 1 activity	molecular function	0.061458529
GO:0010815	bradykinin catabolic process	biological process	0.061458529
GO:0010922	positive regulation of phosphatase activity	biological process	0.061458529
GO:0010923	negative regulation of phosphatase activity	biological process	0.061458529
GO:0016264	gap junction assembly	biological process	0.061458529
GO:0019828	aspartic-type endopeptidase inhibitor activity	molecular function	0.061458529
GO:0020005	symbiont-containing vacuole membrane	cellular component	0.061458529
GO:0031726	CCR1 chemokine receptor binding	molecular function	0.061458529
GO:0031727	CCR2 chemokine receptor binding	molecular function	0.061458529
GO:0032713	negative regulation of interleukin-4 production	biological process	0.061458529
GO:0032714	negative regulation of interleukin-5 production	biological process	0.061458529
GO:0033080	immature T cell proliferation in thymus	biological process	0.061458529
GO:0033092	ositive regulation of immature T cell proliferation in thymu	biological process	0.061458529
GO:0035425	autocrine signaling	biological process	0.061458529
GO:0042490	mechanoreceptor differentiation	biological process	0.061458529
GO:0043372	positive regulation of CD4-positive, alpha-beta T cell differentiation	biological process	0.061458529
GO:0045836	positive regulation of meiotic nuclear division	biological process	0.061458529
GO:0045959	negative regulation of complement activation, classical pathway	biological process	0.061458529
GO:0050672	negative regulation of lymphocyte proliferation	biological process	0.061458529
GO:0050917	sensory perception of umami taste	biological process	0.061458529
GO:0050957	equilibrioception	biological process	0.061458529
GO:0051707	response to other organism	biological process	0.061458529
GO:0060028	convergent extension involved in axis elongation	biological process	0.061458529
GO:0060669	embryonic placenta morphogenesis	biological process	0.061458529
GO:0070318	positive regulation of G0 to G1 transition	biological process	0.061458529

GO:0070831	basement membrane assembly	biological process	0.061458529
GO:0090559	regulation of membrane permeability	biological process	0.061458529
GO:0120200	rod photoreceptor outer segment	cellular component	0.061458529
GO:1902952	positive regulation of dendritic spine maintenance	biological process	0.061458529
GO:1903348	positive regulation of bicellular tight junction assembly	biological process	0.061458529
GO:2001269	of cysteine-type endopeptidase activity involved in apoptoti	biological process	0.061458529
GO:0097192	extrinsic apoptotic signaling pathway in absence of ligand	biological process	0.062219043
GO:0004930	G protein-coupled receptor activity	molecular function	0.062541864
GO:0060070	canonical Wnt signaling pathway	biological process	0.063397544
GO:0004869	cysteine-type endopeptidase inhibitor activity	molecular function	0.064672397
GO:0001503	ossification	biological process	0.066424468
GO:0046330	positive regulation of JNK cascade	biological process	0.066424468
GO:0006936	muscle contraction	biological process	0.069674608
GO:0002232	leukocyte chemotaxis involved in inflammatory response	biological process	0.06992603
GO:0002674	negative regulation of acute inflammatory response	biological process	0.06992603
GO:0002752	cell surface pattern recognition receptor signaling pathway	biological process	0.06992603
GO:0002933	lipid hydroxylation	biological process	0.06992603
GO:0004568	chitinase activity	molecular function	0.06992603
GO:0005031	tumor necrosis factor receptor activity	molecular function	0.06992603
GO:0005664	nuclear origin of replication recognition complex	cellular component	0.06992603
GO:0006081	cellular aldehyde metabolic process	biological process	0.06992603
GO:0010518	positive regulation of phospholipase activity	biological process	0.06992603
GO:0010837	regulation of keratinocyte proliferation	biological process	0.06992603
GO:0016818	hydrolase activity, acting on acid anhydrides, in phosphorus-containing anhydrides	molecular function	0.06992603
GO:0021520	spinal cord motor neuron cell fate specification	biological process	0.06992603
GO:0030223	neutrophil differentiation	biological process	0.06992603
GO:0032730	positive regulation of interleukin-1 alpha production	biological process	0.06992603
GO:0034774	secretory granule lumen	cellular component	0.06992603
GO:0035173	histone kinase activity	molecular function	0.06992603
GO:0038036	sphingosine-1-phosphate receptor activity	molecular function	0.06992603
GO:0043139	5'-3' DNA helicase activity	molecular function	0.06992603
GO:0048318	axial mesoderm development	biological process	0.06992603
GO:0048743	positive regulation of skeletal muscle fiber development	biological process	0.06992603
GO:0048806	genitalia development	biological process	0.06992603
GO:0048874	ost-mediated regulation of intestinal microbiota compositio	biological process	0.06992603
GO:0050915	sensory perception of sour taste	biological process	0.06992603
GO:0051026	chiasma assembly	biological process	0.06992603
GO:0051147	regulation of muscle cell differentiation	biological process	0.06992603
GO:0051353	positive regulation of oxidoreductase activity	biological process	0.06992603
GO:0051461	positive regulation of corticotropin secretion	biological process	0.06992603
GO:0070891	lipoteichoic acid binding	molecular function	0.06992603
GO:0090175	regulation of establishment of planar polarity	biological process	0.06992603
GO:0090312	positive regulation of protein deacetylation	biological process	0.06992603
GO:0090324	negative regulation of oxidative phosphorylation	biological process	0.06992603
GO:0097300	programmed necrotic cell death	biological process	0.06992603
GO:2000253	positive regulation of feeding behavior	biological process	0.06992603
GO:0008201	heparin binding	molecular function	0.070579705
GO:0003697	single-stranded DNA binding	molecular function	0.071086729
GO:0008150	biological process	biological process	0.073945819

GO:0007193	adenylate cyclase-inhibiting G protein-coupled receptor signaling pathway	biological process	0.074799073
GO:0030335	positive regulation of cell migration	biological process	0.075456363
GO:0009617	response to bacterium	biological process	0.076611481
GO:0009749	response to glucose	biological process	0.077405283
GO:0002446	neutrophil mediated immunity	biological process	0.078317541
GO:0003689	DNA clamp loader activity	molecular function	0.078317541
GO:0003810	protein-glutamine gamma-glutamyltransferase activity	molecular function	0.078317541
GO:0004028	3-chloroallyl aldehyde dehydrogenase activity	molecular function	0.078317541
GO:0004298	threonine-type endopeptidase activity	molecular function	0.078317541
GO:0006032	chitin catabolic process	biological process	0.078317541
GO:0006924	activation-induced cell death of T cells	biological process	0.078317541
GO:0008278	cohesin complex	cellular component	0.078317541
GO:0010818	T cell chemotaxis	biological process	0.078317541
GO:0010997	anaphase-promoting complex binding	molecular function	0.078317541
GO:0022898	regulation of transmembrane transporter activity	biological process	0.078317541
GO:0030983	mismatched DNA binding	molecular function	0.078317541
GO:0031017	exocrine pancreas development	biological process	0.078317541
GO:0032494	response to peptidoglycan	biological process	0.078317541
GO:0034875	caffeine oxidase activity	molecular function	0.078317541
GO:0042118	endothelial cell activation	biological process	0.078317541
GO:0043184	vascular endothelial growth factor receptor 2 binding	molecular function	0.078317541
GO:0045953	egative regulation of natural killer cell mediated cytotoxicit	biological process	0.078317541
GO:0046620	regulation of organ growth	biological process	0.078317541
GO:0047704	bile-salt sulfotransferase activity	molecular function	0.078317541
GO:0048935	peripheral nervous system neuron development	biological process	0.078317541
GO:0050649	testosterone 6-beta-hydroxylase activity	molecular function	0.078317541
GO:0051880	G-quadruplex DNA binding	molecular function	0.078317541
GO:0060054	regulation of epithelial cell proliferation involved in wound	biological process	0.078317541
GO:0060117	auditory receptor cell development	biological process	0.078317541
GO:0070493	thrombin-activated receptor signaling pathway	biological process	0.078317541
GO:0097250	mitochondrial respirasome assembly	biological process	0.078317541
GO:2000320	negative regulation of T-helper 17 cell differentiation	biological process	0.078317541
GO:0000902	cell morphogenesis	biological process	0.079173826
GO:0050829	defense response to Gram-negative bacterium	biological process	0.079173826
GO:0002227	innate immune response in mucosa	biological process	0.08003984
GO:0042130	negative regulation of T cell proliferation	biological process	0.08003984
GO:0045095	keratin filament	cellular component	0.08003984
GO:0007189	te cyclase-activating G protein-coupled receptor signaling p	biological process	0.08083758
GO:0050728	negative regulation of inflammatory response	biological process	0.08083758
GO:0042742	defense response to bacterium	biological process	0.081617824
GO:0016042	lipid catabolic process	biological process	0.082516469
GO:0007165	signal transduction	biological process	0.082811811
GO:0006508	proteolysis	biological process	0.0830494
GO:0007254	JNK cascade	biological process	0.085391133
GO:0032722	positive regulation of chemokine production	biological process	0.085391133
GO:0008233	peptidase activity	molecular function	0.085410869
GO:0006260	DNA replication	biological process	0.085919106
GO:0002637	regulation of immunoglobulin production	biological process	0.086633739
GO:0004027	alcohol sulfotransferase activity	molecular function	0.086633739
GO:0004382	GDP phosphatase activity	molecular function	0.086633739

GO:0007292	female gamete generation	biological process	0.086633739
GO:0008020	G protein-coupled photoreceptor activity	molecular function	0.086633739
GO:0009435	NAD biosynthetic process	biological process	0.086633739
GO:0009881	photoreceptor activity	molecular function	0.086633739
GO:0010944	negative regulation of transcription by competitive promoter binding	biological process	0.086633739
GO:0014883	transition between fast and slow fiber	biological process	0.086633739
GO:0016410	N-acyltransferase activity	molecular function	0.086633739
GO:0019363	pyridine nucleotide biosynthetic process	biological process	0.086633739
GO:0019774	proteasome core complex, beta-subunit complex	cellular component	0.086633739
GO:0030056	hemidesmosome	cellular component	0.086633739
GO:0030070	insulin processing	biological process	0.086633739
GO:0030125	clathrin vesicle coat	cellular component	0.086633739
GO:0043367	CD4-positive, alpha-beta T cell differentiation	biological process	0.086633739
GO:0043878	glyceraldehyde-3-phosphate dehydrogenase (NAD+) (non-phosphorylating) activity	molecular function	0.086633739
GO:0045134	UDP phosphatase activity	molecular function	0.086633739
GO:0045617	negative regulation of keratinocyte differentiation	biological process	0.086633739
GO:0047044	androstan-3-alpha,17-beta-diol dehydrogenase activity	molecular function	0.086633739
GO:0047499	calcium-independent phospholipase A2 activity	molecular function	0.086633739
GO:0050544	arachidonic acid binding	molecular function	0.086633739
GO:0051093	negative regulation of developmental process	biological process	0.086633739
GO:0051148	negative regulation of muscle cell differentiation	biological process	0.086633739
GO:0060736	prostate gland growth	biological process	0.086633739
GO:0070314	G1 to G0 transition	biological process	0.086633739
GO:0071223	cellular response to lipoteichoic acid	biological process	0.086633739
GO:0071467	cellular response to pH	biological process	0.086633739
GO:0072378	blood coagulation, fibrin clot formation	biological process	0.086633739
GO:0099503	secretory vesicle	cellular component	0.086633739
GO:0101021	estrogen 2-hydroxylase activity	molecular function	0.086633739
GO:0120199	cone photoreceptor outer segment	cellular component	0.086633739
GO:0016573	histone acetylation	biological process	0.088106468
GO:0045111	intermediate filament cytoskeleton	cellular component	0.088106468
GO:0005201	extracellular matrix structural constituent	molecular function	0.089380638
GO:0042127	regulation of cell population proliferation	biological process	0.09075751
GO:0030178	negative regulation of Wnt signaling pathway	biological process	0.090847344
GO:0030514	negative regulation of BMP signaling pathway	biological process	0.090847344
GO:0042311	vasodilation	biological process	0.090847344
GO:1902476	chloride transmembrane transport	biological process	0.090847344
GO:0032496	response to lipopolysaccharide	biological process	0.091133142
GO:0004867	serine-type endopeptidase inhibitor activity	molecular function	0.092899949
GO:0002262	myeloid cell homeostasis	biological process	0.094875297
GO:0002819	regulation of adaptive immune response	biological process	0.094875297
GO:0005237	inhibitory extracellular ligand-gated monoatomic ion channel activity	molecular function	0.094875297
GO:0005513	detection of calcium ion	biological process	0.094875297
GO:0006957	complement activation, alternative pathway	biological process	0.094875297
GO:0006983	ER overload response	biological process	0.094875297
GO:0007127	meiosis I	biological process	0.094875297
GO:0008061	chitin binding	molecular function	0.094875297
GO:0008503	benzodiazepine receptor activity	molecular function	0.094875297
GO:0010940	positive regulation of necrotic cell death	biological process	0.094875297

GO:0016075	rRNA catabolic process	biological process	0.094875297
GO:0035067	negative regulation of histone acetylation	biological process	0.094875297
GO:0038180	nerve growth factor signaling pathway	biological process	0.094875297
GO:0042379	chemokine receptor binding	molecular function	0.094875297
GO:0042759	long-chain fatty acid biosynthetic process	biological process	0.094875297
GO:0045277	respiratory chain complex IV	cellular component	0.094875297
GO:0046651	lymphocyte proliferation	biological process	0.094875297
GO:0048762	mesenchymal cell differentiation	biological process	0.094875297
GO:0050656	3'-phosphoadenosine 5'-phosphosulfate binding	molecular function	0.094875297
GO:0050786	RAGE receptor binding	molecular function	0.094875297
GO:0051602	response to electrical stimulus	biological process	0.094875297
GO:0060314	regulation of ryanodine-sensitive calcium-release channel activity	biological process	0.094875297
GO:0070410	co-SMAD binding	molecular function	0.094875297
GO:0090385	phagosome-lysosome fusion	biological process	0.094875297
GO:0090571	RNA polymerase II transcription repressor complex	cellular component	0.094875297
GO:1901380	negative regulation of potassium ion transmembrane transport	biological process	0.094875297
GO:1902035	positive regulation of hematopoietic stem cell proliferation	biological process	0.094875297
GO:1902093	positive regulation of flagellated sperm motility	biological process	0.094875297
GO:1903715	regulation of aerobic respiration	biological process	0.094875297
GO:1990440	positive regulation of transcription from RNA polymerase II promoter in response to endoplasmic reticulum stress	biological process	0.094875297
GO:2000242	negative regulation of reproductive process	biological process	0.094875297
GO:2000318	positive regulation of T-helper 17 type immune response	biological process	0.094875297
GO:0008630	insic apoptotic signaling pathway in response to DNA dam	biological process	0.096403027
GO:0003151	outflow tract morphogenesis	biological process	0.099216513
GO:0019902	phosphatase binding	molecular function	0.099216513
GO:0002690	positive regulation of leukocyte chemotaxis	biological process	0.10304288
GO:0004468	lysine N-acetyltransferase activity, acting on acetyl phosphate as donor	molecular function	0.10304288
GO:0006977	ponse, signal transduction by p53 class mediator resulting i	biological process	0.10304288
GO:0009913	epidermal cell differentiation	biological process	0.10304288
GO:0015269	calcium-activated potassium channel activity	molecular function	0.10304288
GO:0017110	nucleoside diphosphate phosphatase activity	molecular function	0.10304288
GO:0032495	response to muramyl dipeptide	biological process	0.10304288
GO:0034638	phosphatidylcholine catabolic process	biological process	0.10304288
GO:0035739	CD4-positive, alpha-beta T cell proliferation	biological process	0.10304288
GO:0043030	regulation of macrophage activation	biological process	0.10304288
GO:0050927	positive regulation of positive chemotaxis	biological process	0.10304288
GO:0050982	detection of mechanical stimulus	biological process	0.10304288
GO:0051400	BH domain binding	molecular function	0.10304288
GO:0070201	regulation of establishment of protein localization	biological process	0.10304288
GO:0071361	cellular response to ethanol	biological process	0.10304288
GO:0071468	cellular response to acidic pH	biological process	0.10304288
GO:0090557	establishment of endothelial intestinal barrier	biological process	0.10304288
GO:0140713	histone chaperone activity	molecular function	0.10304288
GO:1903028	positive regulation of opsonization	biological process	0.10304288
GO:1990535	neuron projection maintenance	biological process	0.10304288
GO:2000273	positive regulation of signaling receptor activity	biological process	0.10304288

GO:2000562	negative regulation of CD4-positive, alpha-beta T cell proliferation	biological process	0.10304288
GO:0005815	microtubule organizing center	cellular component	0.104911542
GO:0001917	photoreceptor inner segment	cellular component	0.107791824
GO:0001947	heart looping	biological process	0.107791824
GO:0008544	epidermis development	biological process	0.107791824
GO:0031965	nuclear membrane	cellular component	0.108960894
GO:0004129	cytochrome-c oxidase activity	molecular function	0.111137151
GO:0004865	protein serine/threonine phosphatase inhibitor activity	molecular function	0.111137151
GO:0004875	complement receptor activity	molecular function	0.111137151
GO:0010459	negative regulation of heart rate	biological process	0.111137151
GO:0010485	histone H4 acetyltransferase activity	molecular function	0.111137151
GO:0017111	ribonucleoside triphosphate phosphatase activity	molecular function	0.111137151
GO:0018022	peptidyl-lysine methylation	biological process	0.111137151
GO:0022851	GABA-gated chloride ion channel activity	molecular function	0.111137151
GO:0032009	early phagosome	cellular component	0.111137151
GO:0032613	interleukin-10 production	biological process	0.111137151
GO:0032675	regulation of interleukin-6 production	biological process	0.111137151
GO:0033162	melanosome membrane	cellular component	0.111137151
GO:0033689	negative regulation of osteoblast proliferation	biological process	0.111137151
GO:0034145	positive regulation of toll-like receptor 4 signaling pathway	biological process	0.111137151
GO:0034374	low-density lipoprotein particle remodeling	biological process	0.111137151
GO:0035456	response to interferon-beta	biological process	0.111137151
GO:0043268	positive regulation of potassium ion transport	biological process	0.111137151
GO:0045618	positive regulation of keratinocyte differentiation	biological process	0.111137151
GO:0046549	retinal cone cell development	biological process	0.111137151
GO:0050687	negative regulation of defense response to virus	biological process	0.111137151
GO:0050700	CARD domain binding	molecular function	0.111137151
GO:0050953	sensory perception of light stimulus	biological process	0.111137151
GO:0060394	negative regulation of pathway-restricted SMAD protein phosphorylation	biological process	0.111137151
GO:0060429	epithelium development	biological process	0.111137151
GO:0072687	meiotic spindle	cellular component	0.111137151
GO:1900264	positive regulation of DNA-directed DNA polymerase activity	biological process	0.111137151
GO:1990806	ligand-gated ion channel signaling pathway	biological process	0.111137151
GO:2000252	negative regulation of feeding behavior	biological process	0.111137151
GO:0009566	fertilization	biological process	0.113614831
GO:0002082	regulation of oxidative phosphorylation	biological process	0.119158763
GO:0003823	antigen binding	molecular function	0.119158763
GO:0005227	calcium activated cation channel activity	molecular function	0.119158763
GO:0005865	striated muscle thin filament	cellular component	0.119158763
GO:0005940	septin ring	cellular component	0.119158763
GO:0006790	sulfur compound metabolic process	biological process	0.119158763
GO:0006941	striated muscle contraction	biological process	0.119158763
GO:0010389	regulation of G2/M transition of mitotic cell cycle	biological process	0.119158763
GO:0010717	regulation of epithelial to mesenchymal transition	biological process	0.119158763
GO:0010820	positive regulation of T cell chemotaxis	biological process	0.119158763
GO:0016493	C-C chemokine receptor activity	molecular function	0.119158763
GO:0030510	regulation of BMP signaling pathway	biological process	0.119158763
GO:0031105	septin complex	cellular component	0.119158763
GO:0046622	positive regulation of organ growth	biological process	0.119158763

GO:0046686	response to cadmium ion	biological process	0.119158763
GO:0050777	negative regulation of immune response	biological process	0.119158763
GO:0051371	muscle alpha-actinin binding	molecular function	0.119158763
GO:0060413	atrial septum morphogenesis	biological process	0.119158763
GO:0060993	kidney morphogenesis	biological process	0.119158763
GO:0071482	cellular response to light stimulus	biological process	0.119158763
GO:0090179	planar cell polarity pathway involved in neural tube closure	biological process	0.119158763
GO:0099105	ion channel modulating, G protein-coupled receptor signaling pathway	biological process	0.119158763
GO:1990000	amyloid fibril formation	biological process	0.119158763
GO:2000146	negative regulation of cell motility	biological process	0.119158763
GO:2000178	negative regulation of neural precursor cell proliferation	biological process	0.119158763
GO:0007249	I-kappaB kinase/NF-kappaB signaling	biological process	0.119517089
GO:0032720	negative regulation of tumor necrosis factor production	biological process	0.119517089
GO:0015630	microtubule cytoskeleton	cellular component	0.123990524
GO:0000245	spliceosomal complex assembly	biological process	0.127108364
GO:0002526	acute inflammatory response	biological process	0.127108364
GO:0004198	calcium-dependent cysteine-type endopeptidase activity	molecular function	0.127108364
GO:0006518	peptide metabolic process	biological process	0.127108364
GO:0010744	positive regulation of macrophage derived foam cell differentiation	biological process	0.127108364
GO:0016790	thiolester hydrolase activity	molecular function	0.127108364
GO:0032153	cell division site	cellular component	0.127108364
GO:0034703	cation channel complex	cellular component	0.127108364
GO:0042438	melanin biosynthetic process	biological process	0.127108364
GO:0043508	negative regulation of JUN kinase activity	biological process	0.127108364
GO:0047498	calcium-dependent phospholipase A2 activity	molecular function	0.127108364
GO:0050684	regulation of mRNA processing	biological process	0.127108364
GO:0060347	heart trabecula formation	biological process	0.127108364
GO:0097190	apoptotic signaling pathway	biological process	0.128508864
GO:0006915	apoptotic process	biological process	0.131039827
GO:0016529	sarcoplasmic reticulum	cellular component	0.134589064
GO:0001706	endoderm formation	biological process	0.134986599
GO:0004029	aldehyde dehydrogenase (NAD+) activity	molecular function	0.134986599
GO:0005243	gap junction channel activity	molecular function	0.134986599
GO:0007062	sister chromatid cohesion	biological process	0.134986599
GO:0007064	mitotic sister chromatid cohesion	biological process	0.134986599
GO:0014002	astrocyte development	biological process	0.134986599
GO:0016010	dystrophin-associated glycoprotein complex	cellular component	0.134986599
GO:0030502	negative regulation of bone mineralization	biological process	0.134986599
GO:0031145	anaphase-promoting complex-dependent catabolic process	biological process	0.134986599
GO:0032680	regulation of tumor necrosis factor production	biological process	0.134986599
GO:0035970	peptidyl-threonine dephosphorylation	biological process	0.134986599
GO:0042589	zymogen granule membrane	cellular component	0.134986599
GO:0043046	DNA methylation involved in gamete generation	biological process	0.134986599
GO:0060100	positive regulation of phagocytosis, engulfment	biological process	0.134986599
GO:0060259	regulation of feeding behavior	biological process	0.134986599
GO:0061640	cytoskeleton-dependent cytokinesis	biological process	0.134986599
GO:0061760	antifungal innate immune response	biological process	0.134986599
GO:0070885	negative regulation of calcineurin-NFAT signaling cascade	biological process	0.134986599
GO:0070989	oxidative demethylation	biological process	0.134986599

GO:0140664	ATP-dependent DNA damage sensor activity	molecular function	0.134986599
GO:1904659	glucose transmembrane transport	biological process	0.134986599
GO:1990226	histone methyltransferase binding	molecular function	0.134986599
GO:0019731	antibacterial humoral response	biological process	0.137653229
GO:0009986	cell surface	cellular component	0.139866693
GO:0032922	circadian regulation of gene expression	biological process	0.140732729
GO:0005622	intracellular anatomical structure	cellular component	0.141291123
GO:0003222	ventricular trabecula myocardium morphogenesis	biological process	0.142794104
GO:0006024	glycosaminoglycan biosynthetic process	biological process	0.142794104
GO:0006476	protein deacetylation	biological process	0.142794104
GO:0008395	steroid hydroxylase activity	molecular function	0.142794104
GO:0032633	interleukin-4 production	biological process	0.142794104
GO:0033116	endoplasmic reticulum-Golgi intermediate compartment membrane	cellular component	0.142794104
GO:0033391	chromatoid body	cellular component	0.142794104
GO:0035497	cAMP response element binding	molecular function	0.142794104
GO:0043186	P granule	cellular component	0.142794104
GO:0045820	negative regulation of glycolytic process	biological process	0.142794104
GO:0048066	developmental pigmentation	biological process	0.142794104
GO:0055089	fatty acid homeostasis	biological process	0.142794104
GO:0060766	negative regulation of androgen receptor signaling pathway	biological process	0.142794104
GO:0006006	glucose metabolic process	biological process	0.143827042
GO:0001819	positive regulation of cytokine production	biological process	0.146935654
GO:0006812	monoatomic cation transport	biological process	0.146935654
GO:0008083	growth factor activity	molecular function	0.149700061
GO:0000145	exocyst	cellular component	0.150531511
GO:0008028	monocarboxylic acid transmembrane transporter activity	molecular function	0.150531511
GO:0010880	regulation of release of sequestered calcium ion into cytosol by sarcoplasmic reticulum	biological process	0.150531511
GO:0015718	monocarboxylic acid transport	biological process	0.150531511
GO:0019001	guanyl nucleotide binding	molecular function	0.150531511
GO:0030140	trans-Golgi network transport vesicle	cellular component	0.150531511
GO:0031430	M band	cellular component	0.150531511
GO:0042325	regulation of phosphorylation	biological process	0.150531511
GO:0043295	glutathione binding	molecular function	0.150531511
GO:0045198	establishment of epithelial cell apical/basal polarity	biological process	0.150531511
GO:0045475	locomotor rhythm	biological process	0.150531511
GO:0045591	positive regulation of regulatory T cell differentiation	biological process	0.150531511
GO:0047023	androsterone dehydrogenase activity	molecular function	0.150531511
GO:0050708	regulation of protein secretion	biological process	0.150531511
GO:0090280	positive regulation of calcium ion import	biological process	0.150531511
GO:0097049	motor neuron apoptotic process	biological process	0.150531511
GO:0098662	inorganic cation transmembrane transport	biological process	0.150531511
GO:0101020	estrogen 16-alpha-hydroxylase activity	molecular function	0.150531511
GO:1903427	negative regulation of reactive oxygen species biosynthetic process	biological process	0.150531511
GO:2000042	negative regulation of double-strand break repair via homologous recombination	biological process	0.150531511
GO:0001664	G protein-coupled receptor binding	molecular function	0.153193762
GO:0006919	activation of cysteine-type endopeptidase activity involved	biological process	0.153193762

GO:0045766	positive regulation of angiogenesis	biological process	0.156110404
GO:0007338	single fertilization	biological process	0.15634227
GO:0016324	apical plasma membrane	cellular component	0.156672865
GO:0000800	lateral element	cellular component	0.158199445
GO:0002177	manchette	cellular component	0.158199445
GO:0002281	macrophage activation involved in immune response	biological process	0.158199445
GO:0002523	leukocyte migration involved in inflammatory response	biological process	0.158199445
GO:0004890	GABA-A receptor activity	molecular function	0.158199445
GO:0005839	proteasome core complex	cellular component	0.158199445
GO:0021675	nerve development	biological process	0.158199445
GO:0022408	negative regulation of cell-cell adhesion	biological process	0.158199445
GO:0032700	negative regulation of interleukin-17 production	biological process	0.158199445
GO:0035094	response to nicotine	biological process	0.158199445
GO:0035909	aorta morphogenesis	biological process	0.158199445
GO:0045745	positive regulation of G protein-coupled receptor signaling pathway	biological process	0.158199445
GO:0060037	pharyngeal system development	biological process	0.158199445
GO:0070266	necroptotic process	biological process	0.158199445
GO:0070628	proteasome binding	molecular function	0.158199445
GO:0071711	basement membrane organization	biological process	0.158199445
GO:0098656	monoatomic anion transmembrane transport	biological process	0.158199445
GO:1902711	GABA-A receptor complex	cellular component	0.158199445
GO:0004519	endonuclease activity	molecular function	0.159503102
GO:0005102	signaling receptor binding	molecular function	0.161738978
GO:0006351	DNA-templated transcription	biological process	0.162605029
GO:0002042	cell migration involved in sprouting angiogenesis	biological process	0.165798529
GO:0003203	endocardial cushion morphogenesis	biological process	0.165798529
GO:0005922	connexin complex	cellular component	0.165798529
GO:0006691	leukotriene metabolic process	biological process	0.165798529
GO:0008401	retinoic acid 4-hydroxylase activity	molecular function	0.165798529
GO:0010804	negative regulation of tumor necrosis factor-mediated signaling pathway	biological process	0.165798529
GO:0014902	myotube differentiation	biological process	0.165798529
GO:0030050	vesicle transport along actin filament	biological process	0.165798529
GO:0031290	retinal ganglion cell axon guidance	biological process	0.165798529
GO:0032623	interleukin-2 production	biological process	0.165798529
GO:0043034	costamere	cellular component	0.165798529
GO:0045104	intermediate filament cytoskeleton organization	biological process	0.165798529
GO:0045178	basal part of cell	cellular component	0.165798529
GO:0048546	digestive tract morphogenesis	biological process	0.165798529
GO:0051056	regulation of small GTPase mediated signal transduction	biological process	0.165798529
GO:0071577	zinc ion transmembrane transport	biological process	0.165798529
GO:1905564	positive regulation of vascular endothelial cell proliferation	biological process	0.165798529
GO:0008237	metallopeptidase activity	molecular function	0.166979588
GO:0006909	phagocytosis	biological process	0.16905483
GO:0042803	protein homodimerization activity	molecular function	0.172912516
GO:0004950	chemokine receptor activity	molecular function	0.173329375
GO:0006298	mismatch repair	biological process	0.173329375
GO:0016279	protein-lysine N-methyltransferase activity	molecular function	0.173329375
GO:0017116	single-stranded DNA helicase activity	molecular function	0.173329375
GO:0019825	oxygen binding	molecular function	0.173329375

GO:0019897	extrinsic component of plasma membrane	cellular component	0.173329375
GO:0031045	dense core granule	cellular component	0.173329375
GO:0033017	sarcoplasmic reticulum membrane	cellular component	0.173329375
GO:0034587	piRNA processing	biological process	0.173329375
GO:0035234	ectopic germ cell programmed cell death	biological process	0.173329375
GO:0043032	positive regulation of macrophage activation	biological process	0.173329375
GO:0047617	acyl-CoA hydrolase activity	molecular function	0.173329375
GO:0048245	eosinophil chemotaxis	biological process	0.173329375
GO:0050709	negative regulation of protein secretion	biological process	0.173329375
GO:0051393	alpha-actinin binding	molecular function	0.173329375
GO:0051591	response to cAMP	biological process	0.173329375
GO:0051893	regulation of focal adhesion assembly	biological process	0.173329375
GO:0072520	seminiferous tubule development	biological process	0.173329375
GO:0007204	positive regulation of cytosolic calcium ion concentration	biological process	0.175830841
GO:0000987	cis-regulatory region sequence-specific DNA binding	molecular function	0.178700827
GO:0009395	phospholipid catabolic process	biological process	0.180792594
GO:0030215	semaphorin receptor binding	molecular function	0.180792594
GO:0030552	cAMP binding	molecular function	0.180792594
GO:0030902	hindbrain development	biological process	0.180792594
GO:0031731	CCR6 chemokine receptor binding	molecular function	0.180792594
GO:0032693	negative regulation of interleukin-10 production	biological process	0.180792594
GO:0035634	response to stilbenoid	biological process	0.180792594
GO:0043687	post-translational protein modification	biological process	0.180792594
GO:0050832	defense response to fungus	biological process	0.180792594
GO:0050995	negative regulation of lipid catabolic process	biological process	0.180792594
GO:0051302	regulation of cell division	biological process	0.180792594
GO:0051403	stress-activated MAPK cascade	biological process	0.180792594
GO:0090026	positive regulation of monocyte chemotaxis	biological process	0.180792594
GO:0008236	serine-type peptidase activity	molecular function	0.187074118
GO:0005385	zinc ion transmembrane transporter activity	molecular function	0.18818879
GO:0006270	DNA replication initiation	biological process	0.18818879
GO:0008053	mitochondrial fusion	biological process	0.18818879
GO:0008361	regulation of cell size	biological process	0.18818879
GO:0034704	calcium channel complex	cellular component	0.18818879
GO:0045821	positive regulation of glycolytic process	biological process	0.18818879
GO:0046328	regulation of JNK cascade	biological process	0.18818879
GO:0050965	detection of temperature stimulus involved in sensory perception of pain	biological process	0.18818879
GO:0060088	auditory receptor cell stereocilium organization	biological process	0.18818879
GO:0090190	positive regulation of branching involved in ureteric bud morphogenesis	biological process	0.18818879
GO:0097110	scaffold protein binding	molecular function	0.19168842
GO:0001741	XY body	cellular component	0.195518562
GO:0003009	skeletal muscle contraction	biological process	0.195518562
GO:0007099	centriole replication	biological process	0.195518562
GO:0010765	positive regulation of sodium ion transport	biological process	0.195518562
GO:0016209	antioxidant activity	molecular function	0.195518562
GO:0021522	spinal cord motor neuron differentiation	biological process	0.195518562
GO:0030318	melanocyte differentiation	biological process	0.195518562
GO:0030742	GTP-dependent protein binding	molecular function	0.195518562
GO:0031683	G-protein beta/gamma-subunit complex binding	molecular function	0.195518562

GO:0032331	negative regulation of chondrocyte differentiation	biological process	0.195518562
GO:0032874	positive regulation of stress-activated MAPK cascade	biological process	0.195518562
GO:0032930	positive regulation of superoxide anion generation	biological process	0.195518562
GO:0043395	heparan sulfate proteoglycan binding	molecular function	0.195518562
GO:0043505	CENP-A containing nucleosome	cellular component	0.195518562
GO:0048598	embryonic morphogenesis	biological process	0.195518562
GO:0050908	detection of light stimulus involved in visual perception	biological process	0.195518562
GO:0070840	dynein complex binding	molecular function	0.195518562
GO:0071549	cellular response to dexamethasone stimulus	biological process	0.195518562
GO:2000647	negative regulation of stem cell proliferation	biological process	0.195518562
GO:0008284	positive regulation of cell population proliferation	biological process	0.195880752
GO:0008289	lipid binding	molecular function	0.200218508
GO:0007218	neuropeptide signaling pathway	biological process	0.201509314
GO:0071944	cell periphery	cellular component	0.201509314
GO:0003382	epithelial cell morphogenesis	biological process	0.202782503
GO:0006829	zinc ion transport	biological process	0.202782503
GO:0015012	heparan sulfate proteoglycan biosynthetic process	biological process	0.202782503
GO:0016620	oxidoreductase activity, acting on the aldehyde or oxo group of donors, NAD or NADP as acceptor	molecular function	0.202782503
GO:0021983	pituitary gland development	biological process	0.202782503
GO:0032753	positive regulation of interleukin-4 production	biological process	0.202782503
GO:0034113	heterotypic cell-cell adhesion	biological process	0.202782503
GO:0048665	neuron fate specification	biological process	0.202782503
GO:0050482	arachidonic acid secretion	biological process	0.202782503
GO:0050699	WW domain binding	molecular function	0.202782503
GO:0070412	R-SMAD binding	molecular function	0.202782503
GO:0070509	calcium ion import	biological process	0.202782503
GO:0018105	peptidyl-serine phosphorylation	biological process	0.203116296
GO:0032991	protein-containing complex	cellular component	0.20392255
GO:0001649	osteoblast differentiation	biological process	0.20479611
GO:0001702	gastrulation with mouth forming second	biological process	0.209981201
GO:0004745	NAD-retinol dehydrogenase activity	molecular function	0.209981201
GO:0005640	nuclear outer membrane	cellular component	0.209981201
GO:0007342	fusion of sperm to egg plasma membrane involved in single fertilization	biological process	0.209981201
GO:0010575	positive regulation of vascular endothelial growth factor production	biological process	0.209981201
GO:0016540	protein autoprocessing	biological process	0.209981201
GO:0022409	positive regulation of cell-cell adhesion	biological process	0.209981201
GO:0031018	endocrine pancreas development	biological process	0.209981201
GO:0032703	negative regulation of interleukin-2 production	biological process	0.209981201
GO:0035567	non-canonical Wnt signaling pathway	biological process	0.209981201
GO:0055001	muscle cell development	biological process	0.209981201
GO:0072657	protein localization to membrane	biological process	0.209981201
GO:0010008	endosome membrane	cellular component	0.211387168
GO:0016485	protein processing	biological process	0.211387168
GO:0005509	calcium ion binding	molecular function	0.212432768
GO:0001836	release of cytochrome c from mitochondria	biological process	0.217115239
GO:0006958	complement activation, classical pathway	biological process	0.217115239
GO:0008527	taste receptor activity	molecular function	0.217115239
GO:0015267	channel activity	molecular function	0.217115239
GO:0016327	apicolateral plasma membrane	cellular component	0.217115239

GO:0016788	hydrolase activity, acting on ester bonds	molecular function	0.217115239
GO:0032781	positive regulation of ATP-dependent activity	biological process	0.217115239
GO:0036342	post-anal tail morphogenesis	biological process	0.217115239
GO:0042104	positive regulation of activated T cell proliferation	biological process	0.217115239
GO:0042178	xenobiotic catabolic process	biological process	0.217115239
GO:0042573	retinoic acid metabolic process	biological process	0.217115239
GO:0046329	negative regulation of JNK cascade	biological process	0.217115239
GO:0048247	lymphocyte chemotaxis	biological process	0.217115239
GO:0048843	negative regulation of axon extension involved in axon guidance	biological process	0.217115239
GO:0051247	positive regulation of protein metabolic process	biological process	0.217115239
GO:0051259	protein complex oligomerization	biological process	0.217115239
GO:0051262	protein tetramerization	biological process	0.217115239
GO:0060716	labyrinthine layer blood vessel development	biological process	0.217115239
GO:0070160	tight junction	cellular component	0.217115239
GO:2000377	regulation of reactive oxygen species metabolic process	biological process	0.217115239
GO:0003677	DNA binding	molecular function	0.222873265
GO:0031012	extracellular matrix	cellular component	0.223484939
GO:0001675	acrosome assembly	biological process	0.224185194
GO:0003148	outflow tract septum morphogenesis	biological process	0.224185194
GO:0004181	metallocarboxypeptidase activity	molecular function	0.224185194
GO:0004712	protein serine/threonine/tyrosine kinase activity	molecular function	0.224185194
GO:0005184	neuropeptide hormone activity	molecular function	0.224185194
GO:0005657	replication fork	cellular component	0.224185194
GO:0005868	cytoplasmic dynein complex	cellular component	0.224185194
GO:0006368	transcription elongation by RNA polymerase II	biological process	0.224185194
GO:0006882	intracellular zinc ion homeostasis	biological process	0.224185194
GO:0007520	myoblast fusion	biological process	0.224185194
GO:0009880	embryonic pattern specification	biological process	0.224185194
GO:0009954	proximal/distal pattern formation	biological process	0.224185194
GO:0032740	positive regulation of interleukin-17 production	biological process	0.224185194
GO:0044331	cell-cell adhesion mediated by cadherin	biological process	0.224185194
GO:0051384	response to glucocorticoid	biological process	0.224185194
GO:0051932	synaptic transmission, GABAergic	biological process	0.224185194
GO:0055010	ventricular cardiac muscle tissue morphogenesis	biological process	0.224185194
GO:0090303	positive regulation of wound healing	biological process	0.224185194
GO:1903076	regulation of protein localization to plasma membrane	biological process	0.224185194
GO:0046872	metal ion binding	molecular function	0.227703015
GO:0004693	cyclin-dependent protein serine/threonine kinase activity	molecular function	0.23119164
GO:0006637	acyl-CoA metabolic process	biological process	0.23119164
GO:0008210	estrogen metabolic process	biological process	0.23119164
GO:0010971	positive regulation of G2/M transition of mitotic cell cycle	biological process	0.23119164
GO:0032508	DNA duplex unwinding	biological process	0.23119164
GO:0043001	Golgi to plasma membrane protein transport	biological process	0.23119164
GO:0043393	regulation of protein binding	biological process	0.23119164
GO:0045499	chemorepellent activity	molecular function	0.23119164
GO:1902042	negative regulation of extrinsic apoptotic signaling pathway	biological process	0.23119164
GO:0005789	endoplasmic reticulum membrane	cellular component	0.231956963
GO:0001892	embryonic placenta development	biological process	0.238135144
GO:0007214	gamma-aminobutyric acid signaling pathway	biological process	0.238135144

GO:0010592	positive regulation of lamellipodium assembly	biological process	0.238135144
GO:0015908	fatty acid transport	biological process	0.238135144
GO:0032148	activation of protein kinase B activity	biological process	0.238135144
GO:0045089	positive regulation of innate immune response	biological process	0.238135144
GO:0061003	positive regulation of dendritic spine morphogenesis	biological process	0.238135144
GO:0061484	hematopoietic stem cell homeostasis	biological process	0.238135144
GO:0090023	positive regulation of neutrophil chemotaxis	biological process	0.238135144
GO:0006955	immune response	biological process	0.242768494
GO:0004364	glutathione transferase activity	molecular function	0.245016267
GO:0004623	phospholipase A2 activity	molecular function	0.245016267
GO:0006139	nucleobase-containing compound metabolic process	biological process	0.245016267
GO:0008094	ATP-dependent activity, acting on DNA	molecular function	0.245016267
GO:0008157	protein phosphatase 1 binding	molecular function	0.245016267
GO:0017147	Wnt-protein binding	molecular function	0.245016267
GO:0030279	negative regulation of ossification	biological process	0.245016267
GO:0035035	histone acetyltransferase binding	molecular function	0.245016267
GO:0043122	regulation of I-kappaB kinase/NF-kappaB signaling	biological process	0.245016267
GO:0045662	negative regulation of myoblast differentiation	biological process	0.245016267
GO:0048009	insulin-like growth factor receptor signaling pathway	biological process	0.245016267
GO:0048041	focal adhesion assembly	biological process	0.245016267
GO:0048535	lymph node development	biological process	0.245016267
GO:0048741	skeletal muscle fiber development	biological process	0.245016267
GO:0050730	regulation of peptidyl-tyrosine phosphorylation	biological process	0.245016267
GO:0060078	regulation of postsynaptic membrane potential	biological process	0.245016267
GO:0005975	carbohydrate metabolic process	biological process	0.245662957
GO:0032880	regulation of protein localization	biological process	0.251274489
GO:0002092	positive regulation of receptor internalization	biological process	0.251835569
GO:0005272	sodium channel activity	molecular function	0.251835569
GO:0007140	male meiotic nuclear division	biological process	0.251835569
GO:0015026	coreceptor activity	molecular function	0.251835569
GO:0016342	catenin complex	cellular component	0.251835569
GO:0042470	melanosome	cellular component	0.251835569
GO:0043552	positive regulation of phosphatidylinositol 3-kinase activity	biological process	0.251835569
GO:0016787	hydrolase activity	molecular function	0.253214811
GO:0044877	protein-containing complex binding	molecular function	0.254029598
GO:0001708	cell fate specification	biological process	0.258593601
GO:0002548	monocyte chemotaxis	biological process	0.258593601
GO:0003678	DNA helicase activity	molecular function	0.258593601
GO:0005834	heterotrimeric G-protein complex	cellular component	0.258593601
GO:0005921	gap junction	cellular component	0.258593601
GO:0010719	negative regulation of epithelial to mesenchymal transition	biological process	0.258593601
GO:0017157	regulation of exocytosis	biological process	0.258593601
GO:0034332	adherens junction organization	biological process	0.258593601
GO:0048589	developmental growth	biological process	0.258593601
GO:0050681	nuclear androgen receptor binding	molecular function	0.258593601
GO:0051281	positive regulation of release of sequestered calcium ion into cytosol	biological process	0.258593601
GO:0051973	positive regulation of telomerase activity	biological process	0.258593601
GO:0061098	positive regulation of protein tyrosine kinase activity	biological process	0.258593601
GO:2000379	positive regulation of reactive oxygen species metabolic process	biological process	0.258593601

GO:0005198	structural molecule activity	molecular function	0.261295987
GO:0061844	antimicrobial humoral immune response mediated by antimicrobial peptide	biological process	0.261295987
GO:0005543	phospholipid binding	molecular function	0.264637678
GO:0007613	memory	biological process	0.264637678
GO:0050731	positive regulation of peptidyl-tyrosine phosphorylation	biological process	0.264637678
GO:0001782	B cell homeostasis	biological process	0.26529091
GO:0004860	protein kinase inhibitor activity	molecular function	0.26529091
GO:0008080	N-acetyltransferase activity	molecular function	0.26529091
GO:0008198	ferrous iron binding	molecular function	0.26529091
GO:0030332	cyclin binding	molecular function	0.26529091
GO:0043027	cysteine-type endopeptidase inhibitor activity involved in apoptotic process	molecular function	0.26529091
GO:0045746	negative regulation of Notch signaling pathway	biological process	0.26529091
GO:0051497	negative regulation of stress fiber assembly	biological process	0.26529091
GO:0140678	molecular function inhibitor activity	molecular function	0.26529091
GO:0005635	nuclear envelope	cellular component	0.267448637
GO:0005882	intermediate filament	cellular component	0.267979498
GO:0031966	mitochondrial membrane	cellular component	0.267979498
GO:0005604	basement membrane	cellular component	0.271321174
GO:0006874	intracellular calcium ion homeostasis	biological process	0.271321174
GO:0000077	DNA damage checkpoint signaling	biological process	0.27192804
GO:0000146	microfilament motor activity	molecular function	0.27192804
GO:0000307	cyclin-dependent protein kinase holoenzyme complex	cellular component	0.27192804
GO:0018149	peptide cross-linking	biological process	0.27192804
GO:0031297	replication fork processing	biological process	0.27192804
GO:0043388	positive regulation of DNA binding	biological process	0.27192804
GO:0048663	neuron fate commitment	biological process	0.27192804
GO:0070050	neuron cellular homeostasis	biological process	0.27192804
GO:0065003	protein-containing complex assembly	biological process	0.274662439
GO:0000421	autophagosome membrane	cellular component	0.278505528
GO:0001818	negative regulation of cytokine production	biological process	0.278505528
GO:0004521	RNA endonuclease activity	molecular function	0.278505528
GO:0004601	peroxidase activity	molecular function	0.278505528
GO:0010934	macrophage cytokine production	biological process	0.278505528
GO:0019882	antigen processing and presentation	biological process	0.278505528
GO:0043542	endothelial cell migration	biological process	0.278505528
GO:0050919	negative chemotaxis	biological process	0.278505528
GO:0090398	cellular senescence	biological process	0.278505528
GO:0005667	transcription regulator complex	cellular component	0.280349755
GO:0043235	receptor complex	cellular component	0.284547853
GO:0007043	cell-cell junction assembly	biological process	0.285023908
GO:0007095	mitotic G2 DNA damage checkpoint signaling	biological process	0.285023908
GO:0007154	cell communication	biological process	0.285023908
GO:0016339	calcium-dependent cell-cell adhesion via plasma membrane	biological process	0.285023908
GO:0030017	sarcomere	cellular component	0.285023908
GO:0030286	dynein complex	cellular component	0.285023908
GO:0046718	viral entry into host cell	biological process	0.285023908
GO:0060349	bone morphogenesis	biological process	0.285023908
GO:0070330	aromatase activity	molecular function	0.285023908
GO:0090734	site of DNA damage	cellular component	0.285023908

GO:1900182	positive regulation of protein localization to nucleus	biological process	0.285023908
GO:0051897	positive regulation of protein kinase B signaling	biological process	0.288018191
GO:0007155	cell adhesion	biological process	0.288341077
GO:0016491	oxidoreductase activity	molecular function	0.291176908
GO:0000786	nucleosome	cellular component	0.291353545
GO:0030163	protein catabolic process	biological process	0.291353545
GO:0000793	condensed chromosome	cellular component	0.291483707
GO:0008625	extrinsic apoptotic signaling pathway via death domain receptors	biological process	0.291483707
GO:0042832	defense response to protozoan	biological process	0.291483707
GO:0043029	T cell homeostasis	biological process	0.291483707
GO:0043085	positive regulation of catalytic activity	biological process	0.291483707
GO:0045861	negative regulation of proteolysis	biological process	0.291483707
GO:0048701	embryonic cranial skeleton morphogenesis	biological process	0.291483707
GO:0051924	regulation of calcium ion transport	biological process	0.291483707
GO:0071526	semaphorin-plexin signaling pathway	biological process	0.291483707
GO:0043231	intracellular membrane-bounded organelle	cellular component	0.292381049
GO:0071805	potassium ion transmembrane transport	biological process	0.29468698
GO:0000086	G2/M transition of mitotic cell cycle	biological process	0.29788545
GO:0015030	Cajal body	cellular component	0.29788545
GO:0017046	peptide hormone binding	molecular function	0.29788545
GO:0032420	stereocilium	cellular component	0.29788545
GO:0032689	negative regulation of type II interferon production	biological process	0.29788545
GO:0045664	regulation of neuron differentiation	biological process	0.29788545
GO:0055007	cardiac muscle cell differentiation	biological process	0.29788545
GO:0086091	regulation of heart rate by cardiac conduction	biological process	0.29788545
GO:0097730	non-motile cilium	cellular component	0.29788545
GO:0000781	chromosome, telomeric region	cellular component	0.30134715
GO:0004180	carboxypeptidase activity	molecular function	0.304229655
GO:0007163	establishment or maintenance of cell polarity	biological process	0.304229655
GO:0007492	endoderm development	biological process	0.304229655
GO:0008047	enzyme activator activity	molecular function	0.304229655
GO:0031663	lipopolysaccharide-mediated signaling pathway	biological process	0.304229655
GO:0042056	chemoattractant activity	molecular function	0.304229655
GO:0045505	dynein intermediate chain binding	molecular function	0.304229655
GO:0050873	brown fat cell differentiation	biological process	0.304229655
GO:0051539	4 iron, 4 sulfur cluster binding	molecular function	0.304229655
GO:0061436	establishment of skin barrier	biological process	0.304229655
GO:0071345	cellular response to cytokine stimulus	biological process	0.304229655
GO:1905168	positive regulation of double-strand break repair via homologous recombination	biological process	0.304229655
GO:2000781	positive regulation of double-strand break repair	biological process	0.304229655
GO:0004518	nuclease activity	molecular function	0.304673427
GO:0042383	sarcolemma	cellular component	0.304673427
GO:0010629	negative regulation of gene expression	biological process	0.306574661
GO:0001618	virus receptor activity	molecular function	0.310516837
GO:0001965	G-protein alpha-subunit binding	molecular function	0.310516837
GO:0012501	programmed cell death	biological process	0.310516837
GO:0030833	regulation of actin filament polymerization	biological process	0.310516837
GO:0032609	type II interferon production	biological process	0.310516837
GO:0070059	intrinsic apoptotic signaling pathway in response to endoplasmic reticulum stress	biological process	0.310516837

GO:0004222	metalloendopeptidase activity	molecular function	0.311317254
GO:0008202	steroid metabolic process	biological process	0.311317254
GO:0009952	anterior/posterior pattern specification	biological process	0.311317254
GO:0006974	cellular response to DNA damage stimulus	biological process	0.311395643
GO:0006979	response to oxidative stress	biological process	0.314634369
GO:0001816	cytokine production	biological process	0.316747506
GO:0032590	dendrite membrane	cellular component	0.316747506
GO:0040018	positive regulation of multicellular organism growth	biological process	0.316747506
GO:0043536	ositive regulation of blood vessel endothelial cell migratio	biological process	0.316747506
GO:0072593	reactive oxygen species metabolic process	biological process	0.316747506
GO:0007616	long-term memory	biological process	0.322922167
GO:0017124	SH3 domain binding	molecular function	0.324564016
GO:0032735	positive regulation of interleukin-12 production	biological process	0.329041321
GO:0043330	response to exogenous dsRNA	biological process	0.329041321
GO:0051781	positive regulation of cell division	biological process	0.329041321
GO:0051898	negative regulation of protein kinase B signaling	biological process	0.329041321
GO:0000785	chromatin	cellular component	0.330957183
GO:0030018	Z disc	cellular component	0.331163683
GO:0009887	animal organ morphogenesis	biological process	0.334456903
GO:0005504	fatty acid binding	molecular function	0.335105463
GO:0006275	regulation of DNA replication	biological process	0.335105463
GO:0014070	response to organic cyclic compound	biological process	0.335105463
GO:0043473	pigmentation	biological process	0.335105463
GO:0045494	photoreceptor cell maintenance	biological process	0.335105463
GO:0046326	positive regulation of glucose import	biological process	0.335105463
GO:2001237	negative regulation of extrinsic apoptotic signaling pathway	biological process	0.335105463
GO:0002088	lens development in camera-type eye	biological process	0.341115087
GO:0004857	enzyme inhibitor activity	molecular function	0.341115087
GO:0005215	transporter activity	molecular function	0.341115087
GO:0030276	clathrin binding	molecular function	0.341115087
GO:0031640	killing of cells of another organism	biological process	0.341115087
GO:1904315	transmitter-gated monoatomic ion channel activity involved in regulation of postsynaptic membrane potential	molecular function	0.341115087
GO:0006644	phospholipid metabolic process	biological process	0.347070679
GO:0010718	positive regulation of epithelial to mesenchymal transition	biological process	0.347070679
GO:0033077	T cell differentiation in thymus	biological process	0.347070679
GO:0048536	spleen development	biological process	0.347070679
GO:0050909	sensory perception of taste	biological process	0.347070679
GO:0120163	negative regulation of cold-induced thermogenesis	biological process	0.347070679
GO:0007049	cell cycle	biological process	0.347561888
GO:0008234	cysteine-type peptidase activity	molecular function	0.347581376
GO:0001580	detection of chemical stimulus involved in sensory perception of bitter taste	biological process	0.352972723
GO:0007566	embryo implantation	biological process	0.352972723
GO:0016922	nuclear receptor binding	molecular function	0.352972723
GO:0043124	negative regulation of I-kappaB kinase/NF-kappaB signalin	biological process	0.352972723
GO:0060173	limb development	biological process	0.352972723
GO:0071560	ellular response to transforming growth factor beta stimulu	biological process	0.352972723
GO:0003674	molecular function	molecular function	0.354471648
GO:0016567	protein ubiquitination	biological process	0.355542591
GO:0001221	transcription coregulator binding	molecular function	0.358821698

GO:0003727	single-stranded RNA binding	molecular function	0.358821698
GO:0005261	monoatomic cation channel activity	molecular function	0.358821698
GO:0007568	aging	biological process	0.358821698
GO:0030073	insulin secretion	biological process	0.358821698
GO:0051276	chromosome organization	biological process	0.358821698
GO:0004721	phosphoprotein phosphatase activity	molecular function	0.363864971
GO:0001658	branching involved in ureteric bud morphogenesis	biological process	0.364618078
GO:0030331	nuclear estrogen receptor binding	molecular function	0.364618078
GO:0042177	negative regulation of protein catabolic process	biological process	0.364618078
GO:0010628	positive regulation of gene expression	biological process	0.367277597
GO:0005923	bicellular tight junction	cellular component	0.370336247
GO:0009966	regulation of signal transduction	biological process	0.370362334
GO:0030136	clathrin-coated vesicle	cellular component	0.370362334
GO:0031623	receptor internalization	biological process	0.370362334
GO:0034446	substrate adhesion-dependent cell spreading	biological process	0.370362334
GO:0045907	positive regulation of vasoconstriction	biological process	0.370362334
GO:0045177	apical part of cell	cellular component	0.373562235
GO:0005747	mitochondrial respiratory chain complex I	cellular component	0.376054932
GO:0006986	response to unfolded protein	biological process	0.376054932
GO:0030968	endoplasmic reticulum unfolded protein response	biological process	0.376054932
GO:0043539	protein serine/threonine kinase activator activity	molecular function	0.376054932
GO:0005506	iron ion binding	molecular function	0.376781593
GO:0030141	secretory granule	cellular component	0.376781593
GO:0019904	protein domain specific binding	molecular function	0.378040009
GO:0000794	condensed nuclear chromosome	cellular component	0.381696334
GO:0006879	intracellular iron ion homeostasis	biological process	0.381696334
GO:0009953	dorsal/ventral pattern formation	biological process	0.381696334
GO:0030544	Hsp70 protein binding	molecular function	0.381696334
GO:0051289	protein homotetramerization	biological process	0.381696334
GO:0007286	spermatid development	biological process	0.383199856
GO:0015629	actin cytoskeleton	cellular component	0.385322055
GO:0005496	steroid binding	molecular function	0.387286998
GO:0006282	regulation of DNA repair	biological process	0.392827378
GO:0009968	negative regulation of signal transduction	biological process	0.392827378
GO:0030199	collagen fibril organization	biological process	0.392827378
GO:0032715	negative regulation of interleukin-6 production	biological process	0.392827378
GO:0042531	positive regulation of tyrosine phosphorylation of STAT protein	biological process	0.392827378
GO:0048538	thymus development	biological process	0.392827378
GO:0009410	response to xenobiotic stimulus	biological process	0.395950755
GO:0044325	transmembrane transporter binding	molecular function	0.395950755
GO:1903078	positive regulation of protein localization to plasma membrane	biological process	0.398317924
GO:0005178	integrin binding	molecular function	0.399119884
GO:0016887	ATP hydrolysis activity	molecular function	0.399909323
GO:0016712	oxidoreductase activity, acting on paired donors, with incorporation or reduction of molecular oxygen, reduced flavin or flavoprotein as one donor, and incorporation of one atom of oxygen	molecular function	0.403759082
GO:0031397	negative regulation of protein ubiquitination	biological process	0.403759082
GO:0035458	cellular response to interferon-beta	biological process	0.403759082

GO:0043280	positive regulation of cysteine-type endopeptidase activity involved in apoptotic process	biological process	0.403759082
GO:0045668	negative regulation of osteoblast differentiation	biological process	0.403759082
GO:0050877	nervous system process	biological process	0.403759082
GO:0000122	negative regulation of transcription by RNA polymerase II	biological process	0.404097968
GO:0005525	GTP binding	molecular function	0.404533022
GO:0007162	negative regulation of cell adhesion	biological process	0.409151294
GO:0045597	positive regulation of cell differentiation	biological process	0.409151294
GO:0048704	embryonic skeletal system morphogenesis	biological process	0.409151294
GO:0071347	cellular response to interleukin-1	biological process	0.409151294
GO:0016323	basolateral plasma membrane	cellular component	0.409582454
GO:0000775	chromosome, centromeric region	cellular component	0.41171835
GO:0032757	positive regulation of interleukin-8 production	biological process	0.414494997
GO:0046332	SMAD binding	molecular function	0.414494997
GO:0050714	positive regulation of protein secretion	biological process	0.414494997
GO:0070301	cellular response to hydrogen peroxide	biological process	0.414494997
GO:0016829	lyase activity	molecular function	0.417969152
GO:0000502	proteasome complex	cellular component	0.419790626
GO:0030864	cortical actin cytoskeleton	cellular component	0.419790626
GO:0031047	RNA-mediated gene silencing	biological process	0.419790626
GO:0050766	positive regulation of phagocytosis	biological process	0.419790626
GO:0060261	positive regulation of transcription initiation by RNA polymerase II	biological process	0.419790626
GO:0000082	G1/S transition of mitotic cell cycle	biological process	0.430239377
GO:0001533	cornified envelope	cellular component	0.430239377
GO:0010033	response to organic substance	biological process	0.430239377
GO:0030145	manganese ion binding	molecular function	0.430239377
GO:0032981	mitochondrial respiratory chain complex I assembly	biological process	0.430239377
GO:0042552	myelination	biological process	0.430239377
GO:0007507	heart development	biological process	0.431168983
GO:0019722	calcium-mediated signaling	biological process	0.435393349
GO:0030326	embryonic limb morphogenesis	biological process	0.435393349
GO:0050885	neuromuscular process controlling balance	biological process	0.435393349
GO:0043565	sequence-specific DNA binding	molecular function	0.43618528
GO:0007186	G protein-coupled receptor signaling pathway	biological process	0.44519844
GO:0005200	structural constituent of cytoskeleton	molecular function	0.445562579
GO:0009611	response to wounding	biological process	0.445562579
GO:0034097	response to cytokine	biological process	0.445562579
GO:0042100	B cell proliferation	biological process	0.445562579
GO:0009897	external side of plasma membrane	cellular component	0.450295199
GO:0048863	stem cell differentiation	biological process	0.450578663
GO:0003690	double-stranded DNA binding	molecular function	0.454744407
GO:0006281	DNA repair	biological process	0.45481101
GO:0005905	clathrin-coated pit	cellular component	0.455549606
GO:0007200	phospholipase C-activating G protein-coupled receptor signaling pathway	biological process	0.455549606
GO:0007267	cell-cell signaling	biological process	0.455549606
GO:0030971	receptor tyrosine kinase binding	molecular function	0.455549606
GO:0032731	positive regulation of interleukin-1 beta production	biological process	0.455549606
GO:0042098	T cell proliferation	biological process	0.455549606
GO:0051536	iron-sulfur cluster binding	molecular function	0.455549606
GO:0051603	proteolysis involved in protein catabolic process	biological process	0.455549606

GO:0030496	midbody	cellular component	0.457749611
GO:0008344	adult locomotory behavior	biological process	0.46047581
GO:0018107	peptidyl-threonine phosphorylation	biological process	0.46047581
GO:0045296	cadherin binding	molecular function	0.46047581
GO:0070830	bicellular tight junction assembly	biological process	0.46047581
GO:0008285	negative regulation of cell population proliferation	biological process	0.460759571
GO:0032259	methylation	biological process	0.463731392
GO:0045893	positive regulation of DNA-templated transcription	biological process	0.463974646
GO:0030054	cell junction	cellular component	0.464315186
GO:0000149	SNARE binding	molecular function	0.465357677
GO:0005793	endoplasmic reticulum-Golgi intermediate compartment	cellular component	0.465357677
GO:0009615	response to virus	biological process	0.465357677
GO:0010506	regulation of autophagy	biological process	0.465357677
GO:0042110	T cell activation	biological process	0.465357677
GO:0060326	cell chemotaxis	biological process	0.465357677
GO:1901224	positive regulation of NIK/NF-kappaB signaling	biological process	0.465357677
GO:0016746	acyltransferase activity	molecular function	0.466707823
GO:0006334	nucleosome assembly	biological process	0.470195603
GO:0008286	insulin receptor signaling pathway	biological process	0.470195603
GO:0034220	monoatomic ion transmembrane transport	biological process	0.470195603
GO:0007166	cell surface receptor signaling pathway	biological process	0.472631421
GO:0008021	synaptic vesicle	cellular component	0.472631421
GO:0008168	methyltransferase activity	molecular function	0.472631421
GO:0003007	heart morphogenesis	biological process	0.474989982
GO:0007517	muscle organ development	biological process	0.474989982
GO:0015297	antiporter activity	molecular function	0.474989982
GO:0003700	DNA-binding transcription factor activity	molecular function	0.479290539
GO:0015631	tubulin binding	molecular function	0.479741204
GO:0016328	lateral plasma membrane	cellular component	0.479741204
GO:0017018	myosin phosphatase activity	molecular function	0.479741204
GO:0045785	positive regulation of cell adhesion	biological process	0.479741204
GO:0005765	lysosomal membrane	cellular component	0.48435975
GO:0000049	tRNA binding	molecular function	0.484449655
GO:0009925	basal plasma membrane	cellular component	0.484449655
GO:0008092	cytoskeletal protein binding	molecular function	0.489115719
GO:0035861	site of double-strand break	cellular component	0.489115719
GO:0051965	positive regulation of synapse assembly	biological process	0.489115719
GO:0003924	GTPase activity	molecular function	0.491773035
GO:0005581	collagen trimer	cellular component	0.493739774
GO:0008217	regulation of blood pressure	biological process	0.493739774
GO:0031072	heat shock protein binding	molecular function	0.493739774
GO:0045860	positive regulation of protein kinase activity	biological process	0.493739774
GO:0043524	negative regulation of neuron apoptotic process	biological process	0.501648057
GO:0001889	liver development	biological process	0.50286336
GO:0007018	microtubule-based movement	biological process	0.50286336
GO:0019233	sensory perception of pain	biological process	0.50286336
GO:0045665	negative regulation of neuron differentiation	biological process	0.50286336
GO:1990841	promoter-specific chromatin binding	molecular function	0.50286336
GO:0010467	gene expression	biological process	0.50732804
GO:0032436	positive regulation of proteasomal ubiquitin-dependent protein catabolic process	biological process	0.507363634

GO:0008283	cell population proliferation	biological process	0.507556199
GO:0004197	cysteine-type endopeptidase activity	molecular function	0.511823384
GO:0031398	positive regulation of protein ubiquitination	biological process	0.511823384
GO:0045732	positive regulation of protein catabolic process	biological process	0.511823384
GO:0051865	protein autoubiquitination	biological process	0.511823384
GO:0006631	fatty acid metabolic process	biological process	0.515769149
GO:0140297	DNA-binding transcription factor binding	molecular function	0.515769149
GO:0016579	protein deubiquitination	biological process	0.516242974
GO:0016705	oxidoreductase activity, acting on paired donors, with incorporation or reduction of molecular oxygen	molecular function	0.516242974
GO:0032088	egative regulation of NF-kappaB transcription factor activit	biological process	0.516242974
GO:0048661	positive regulation of smooth muscle cell proliferation	biological process	0.516242974
GO:0060041	retina development in camera-type eye	biological process	0.516242974
GO:0004252	serine-type endopeptidase activity	molecular function	0.518561658
GO:0005903	brush border	cellular component	0.520622763
GO:0014068	›ositive regulation of phosphatidylinositol 3-kinase signalin{	biological process	0.520622763
GO:0019003	GDP binding	molecular function	0.520622763
GO:0060348	bone development	biological process	0.520622763
GO:0043410	positive regulation of MAPK cascade	biological process	0.521343507
GO:0004674	protein serine/threonine kinase activity	molecular function	0.522654972
GO:0005902	microvillus	cellular component	0.524963108
GO:0006821	chloride transport	biological process	0.524963108
GO:0032729	positive regulation of type II interferon production	biological process	0.524963108
GO:0050680	negative regulation of epithelial cell proliferation	biological process	0.524963108
GO:0051213	dioxygenase activity	molecular function	0.524963108
GO:0048812	neuron projection morphogenesis	biological process	0.529264362
GO:0004722	protein serine/threonine phosphatase activity	molecular function	0.533526875
GO:0005262	calcium channel activity	molecular function	0.533526875
GO:0007368	determination of left/right symmetry	biological process	0.533526875
GO:0004888	transmembrane signaling receptor activity	molecular function	0.535091645
GO:0001227	DNA-binding transcription repressor activity, RNA polymerase II-specific	molecular function	0.53617995
GO:0051216	cartilage development	biological process	0.537750995
GO:0032869	cellular response to insulin stimulus	biological process	0.546085426
GO:0042060	wound healing	biological process	0.546085426
GO:0043621	protein self-association	molecular function	0.546085426
GO:0050673	epithelial cell proliferation	biological process	0.546085426
GO:0006897	endocytosis	biological process	0.548568538
GO:0005901	caveola	cellular component	0.550196417
GO:0051649	establishment of localization in cell	biological process	0.551231084
GO:0061630	ubiquitin protein ligase activity	molecular function	0.553322809
GO:0005813	centrosome	cellular component	0.557188271
GO:0022008	neurogenesis	biological process	0.558307622
GO:0071356	cellular response to tumor necrosis factor	biological process	0.558307622
GO:0001701	in utero embryonic development	biological process	0.561753478
GO:0000724	double-strand break repair via homologous recombination	biological process	0.562308497
GO:0004725	protein tyrosine phosphatase activity	molecular function	0.562308497
GO:0007179	transforming growth factor beta receptor signaling pathway	biological process	0.562308497
GO:0035725	sodium ion transmembrane transport	biological process	0.562308497
GO:0005758	mitochondrial intermembrane space	cellular component	0.566273323
GO:0030246	carbohydrate binding	molecular function	0.566974838

GO:0000287	magnesium ion binding	molecular function	0.569560058
GO:0000922	spindle pole	cellular component	0.570202423
GO:0016525	negative regulation of angiogenesis	biological process	0.570202423
GO:0004843	cysteine-type deubiquitinase activity	molecular function	0.574096118
GO:0071346	cellular response to type II interferon	biological process	0.574096118
GO:0007160	cell-matrix adhesion	biological process	0.581778555
GO:0050727	regulation of inflammatory response	biological process	0.581778555
GO:0004497	monooxygenase activity	molecular function	0.585567925
GO:0030183	B cell differentiation	biological process	0.585567925
GO:0030509	BMP signaling pathway	biological process	0.585567925
GO:0050729	positive regulation of inflammatory response	biological process	0.589323141
GO:0005911	cell-cell junction	cellular component	0.589841284
GO:0051015	actin filament binding	molecular function	0.589841284
GO:0010468	regulation of gene expression	biological process	0.592501819
GO:0007417	central nervous system development	biological process	0.593044511
GO:0050852	T cell receptor signaling pathway	biological process	0.593044511
GO:0070588	calcium ion transmembrane transport	biological process	0.593044511
GO:0016055	Wnt signaling pathway	biological process	0.594800071
GO:0120162	positive regulation of cold-induced thermogenesis	biological process	0.596732336
GO:0051726	regulation of cell cycle	biological process	0.59971419
GO:0001843	neural tube closure	biological process	0.600386919
GO:0007224	smoothened signaling pathway	biological process	0.600386919
GO:0043679	axon terminus	cellular component	0.604008557
GO:0090630	activation of GTPase activity	biological process	0.604008557
GO:0033138	positive regulation of peptidyl-serine phosphorylation	biological process	0.607597546
GO:0042391	regulation of membrane potential	biological process	0.611154178
GO:0000981	DNA-binding transcription factor activity, RNA polymerase II-specific	molecular function	0.612208292
GO:0008584	male gonad development	biological process	0.614678744
GO:0007169	transmembrane receptor protein tyrosine kinase signaling pathway	biological process	0.61817153
GO:0007156	ıophilic cell adhesion via plasma membrane adhesion molec	biological process	0.621632823
GO:0006366	transcription by RNA polymerase II	biological process	0.623615151
GO:0031594	neuromuscular junction	cellular component	0.625062905
GO:0090263	positive regulation of canonical Wnt signaling pathway	biological process	0.625062905
GO:0005811	lipid droplet	cellular component	0.628462055
GO:0006629	lipid metabolic process	biological process	0.636178288
GO:0060548	negative regulation of cell death	biological process	0.641754852
GO:0005096	GTPase activator activity	molecular function	0.644175458
GO:0030674	protein-macromolecule adaptor activity	molecular function	0.645003458
GO:0007409	axonogenesis	biological process	0.64822276
GO:0034451	centriolar satellite	cellular component	0.64822276
GO:0006887	exocytosis	biological process	0.651413021
GO:0044297	cell body	cellular component	0.651413021
GO:0015293	symporter activity	molecular function	0.654574503
GO:0031410	cytoplasmic vesicle	cellular component	0.657004437
GO:0051260	protein homooligomerization	biological process	0.657707463
GO:0007010	cytoskeleton organization	biological process	0.669959171
GO:0004672	protein kinase activity	molecular function	0.671145858
GO:0030165	PDZ domain binding	molecular function	0.672953316
GO:0004386	helicase activity	molecular function	0.675920442

GO:0001228	DNA-binding transcription activator activity, RNA polymerase II-specific	molecular function	0.678646463
GO:0007219	Notch signaling pathway	biological process	0.67886079
GO:0019900	kinase binding	molecular function	0.67886079
GO:0051087	chaperone binding	molecular function	0.67886079
GO:0051301	cell division	biological process	0.682888546
GO:0006468	protein phosphorylation	biological process	0.689731454
GO:0000166	nucleotide binding	molecular function	0.690544613
GO:0043123	ositive regulation of I-kappaB kinase/NF-kappaB signaling	biological process	0.69316921
GO:0090575	RNA polymerase II transcription regulator complex	cellular component	0.695953868
GO:0019221	cytokine-mediated signaling pathway	biological process	0.698713387
GO:0003682	chromatin binding	molecular function	0.700435174
GO:0003676	nucleic acid binding	molecular function	0.701849633
GO:0005938	cell cortex	cellular component	0.704157908
GO:0030308	negative regulation of cell growth	biological process	0.704157908
GO:0098982	GABA-ergic synapse	cellular component	0.704157908
GO:0001525	angiogenesis	biological process	0.704429386
GO:0006813	potassium ion transport	biological process	0.709504556
GO:0008360	regulation of cell shape	biological process	0.712141726
GO:1990837	sequence-specific double-stranded DNA binding	molecular function	0.71845135
GO:0005777	peroxisome	cellular component	0.722454388
GO:0042826	histone deacetylase binding	molecular function	0.722454388
GO:0005912	adherens junction	cellular component	0.73239949
GO:0006470	protein dephosphorylation	biological process	0.73239949
GO:0016740	transferase activity	molecular function	0.732906629
GO:0019901	protein kinase binding	molecular function	0.735664072
GO:0048471	perinuclear region of cytoplasm	cellular component	0.735747479
GO:0001669	acrosomal vesicle	cellular component	0.737238296
GO:0006816	calcium ion transport	biological process	0.737238296
GO:0005794	Golgi apparatus	cellular component	0.741403575
GO:0001650	fibrillar center	cellular component	0.744333795
GO:0042593	glucose homeostasis	biological process	0.748957914
GO:0048511	rhythmic process	biological process	0.748957914
GO:0001822	kidney development	biological process	0.753498836
GO:0005770	late endosome	cellular component	0.755738565
GO:0020037	heme binding	molecular function	0.75795805
GO:0002020	protease binding	molecular function	0.760157474
GO:0055085	transmembrane transport	biological process	0.760306931
GO:0042802	identical protein binding	molecular function	0.760487013
GO:0008270	zinc ion binding	molecular function	0.761762895
GO:0005634	nucleus	cellular component	0.763256337
GO:0006869	lipid transport	biological process	0.766637176
GO:0007605	sensory perception of sound	biological process	0.772942724
GO:0046982	protein heterodimerization activity	molecular function	0.776483707
GO:0031982	vesicle	cellular component	0.777051977
GO:0007275	multicellular organism development	biological process	0.778529111
GO:0072659	protein localization to plasma membrane	biological process	0.779078777
GO:0002250	adaptive immune response	biological process	0.785049866
GO:0005768	endosome	cellular component	0.786822961
GO:0030027	lamellipodium	cellular component	0.787004336
GO:0005216	monoatomic ion channel activity	molecular function	0.790860402

GO:0005925	focal adhesion	cellular component	0.794647023
GO:0019899	enzyme binding	molecular function	0.795916538
GO:0031175	neuron projection development	biological process	0.796514681
GO:0030198	extracellular matrix organization	biological process	0.802016883
GO:0000977	RNA polymerase II transcription regulatory region sequence-specific DNA binding	molecular function	0.80237866
GO:0022857	transmembrane transporter activity	molecular function	0.805602544
GO:0031514	motile cilium	cellular component	0.805602544
GO:0038023	signaling receptor activity	molecular function	0.807371076
GO:0060090	molecular adaptor activity	molecular function	0.807371076
GO:0036064	ciliary basal body	cellular component	0.809123604
GO:0043547	positive regulation of GTPase activity	biological process	0.809123604
GO:0007411	axon guidance	biological process	0.810860272
GO:0030036	actin cytoskeleton organization	biological process	0.814286595
GO:0016301	kinase activity	molecular function	0.81496293
GO:0046777	protein autophosphorylation	biological process	0.817651175
GO:0005741	mitochondrial outer membrane	cellular component	0.819310657
GO:0016310	phosphorylation	biological process	0.819802906
GO:0016607	nuclear speck	cellular component	0.825589425
GO:0003714	transcription corepressor activity	molecular function	0.825799705
GO:0005654	nucleoplasm	cellular component	0.830216176
GO:0061629	RNA polymerase II-specific DNA-binding transcription factor binding	molecular function	0.832056908
GO:0098609	cell-cell adhesion	biological process	0.832056908
GO:0006914	autophagy	biological process	0.839564909
GO:0005085	guanyl-nucleotide exchange factor activity	molecular function	0.843908455
GO:0043065	positive regulation of apoptotic process	biological process	0.846332671
GO:0005743	mitochondrial inner membrane	cellular component	0.846703197
GO:0003779	actin binding	molecular function	0.855845674
GO:0030182	neuron differentiation	biological process	0.866402017
GO:0005929	cilium	cellular component	0.871446268
GO:0005856	cytoskeleton	cellular component	0.877563422
GO:0030154	cell differentiation	biological process	0.878275469
GO:0045892	negative regulation of DNA-templated transcription	biological process	0.883292644
GO:0043005	neuron projection	cellular component	0.887632511
GO:0004842	ubiquitin-protein transferase activity	molecular function	0.888769741
GO:0005524	ATP binding	molecular function	0.894976256
GO:0016020	membrane	cellular component	0.896609413
GO:0005737	cytoplasm	cellular component	0.900409688
GO:0006357	regulation of transcription by RNA polymerase II	biological process	0.902111369
GO:0008017	microtubule binding	molecular function	0.902169809
GO:0007399	nervous system development	biological process	0.902494984
GO:0098794	postsynapse	cellular component	0.903063062
GO:0000139	Golgi membrane	cellular component	0.903948202
GO:0001934	positive regulation of protein phosphorylation	biological process	0.905694435
GO:0006511	ubiquitin-dependent protein catabolic process	biological process	0.905694435
GO:0016477	cell migration	biological process	0.915528168
GO:0045944	positive regulation of transcription by RNA polymerase II	biological process	0.915752978
GO:0000978	RNA polymerase II cis-regulatory region sequence-specific	molecular function	0.916092076
GO:0005764	lysosome	cellular component	0.920159123

GO:0005769	early endosome	cellular component	0.922227786
GO:0045211	postsynaptic membrane	cellular component	0.923643269
GO:0007283	spermatogenesis	biological process	0.927007331
GO:0005783	endoplasmic reticulum	cellular component	0.928331422
GO:0031267	small GTPase binding	molecular function	0.929053495
GO:0045121	membrane raft	cellular component	0.932859345
GO:0005886	plasma membrane	cellular component	0.938047256
GO:0003723	RNA binding	molecular function	0.949187965
GO:0005515	protein binding	molecular function	0.949240619
GO:0005874	microtubule	cellular component	0.957619437
GO:0006355	regulation of DNA-templated transcription	biological process	0.96547377
GO:0005694	chromosome	cellular component	0.966820757
GO:0035556	intracellular signal transduction	biological process	0.968737919
GO:0006811	monoatomic ion transport	biological process	0.972935446
GO:0005829	cytosol	cellular component	0.974552631
GO:0030424	axon	cellular component	0.983632132
GO:0042995	cell projection	cellular component	0.98621893
GO:0030425	dendrite	cellular component	0.9929002
GO:0005730	nucleolus	cellular component	0.995010281
GO:0015031	protein transport	biological process	0.996724489
GO:0043025	neuronal cell body	cellular component	0.997382578
GO:0005739	mitochondrion	cellular component	0.999363004
GO:0004984	olfactory receptor activity	molecular function	0.999600582
GO:0007608	sensory perception of smell	biological process	0.999655518
GO:0045202	synapse	cellular component	0.999766936
