# Supplementary material for: Effect of 40 Hz light flicker on cognitive impairment and transcriptome of hippocampus in right unilateral common carotid artery occlusion mice
Source: Sci Rep. 2023 Dec 4;13:21361. doi: 10.1038/s41598-023-48897-6 (PMC10695931; doi:10.1038/s41598-023-48897-6)
Supplement: Supplementary file 2 — Supplementary Information 2. [file 41598_2023_48897_MOESM2_ESM.pdf]

Supplementary KEGG enrichment and annotation

ID	Term	Classification level	p-value
mmu00010	Glycolysis / Gluconeogenesis	Metabolism	0.4485627
mmu00120	Primary bile acid biosynthesis	Metabolism	0.1453874
mmu00140	Steroid hormone biosynthesis	Metabolism	0.557466
mmu00190	Oxidative phosphorylation	Metabolism	0.1123427
mmu00230	Purine metabolism	Metabolism	0.6919337
mmu00240	Pyrimidine metabolism	Metabolism	0.3872803
mmu00340	Histidine metabolism	Metabolism	0.2031085
mmu00350	Tyrosine metabolism	Metabolism	0.295008
mmu00360	Phenylalanine metabolism	Metabolism	0.1675009
mmu00380	Tryptophan metabolism	Metabolism	0.3653957
mmu00410	beta-Alanine metabolism	Metabolism	0.2438581
mmu00430	Taurine and hypotaurine metabolism	Metabolism	0.1747458
mmu00480	Glutathione metabolism	Metabolism	0.1291431
mmu00534	Glycosaminoglycan biosynthesis - heparan sulfate / heparin	Metabolism	0.1890495
mmu00564	Glycerophospholipid metabolism	Metabolism	0.5727982
mmu00565	Ether lipid metabolism	Metabolism	0.3369527
mmu00590	Arachidonic acid metabolism	Metabolism	0.1651407
mmu00591	Linoleic acid metabolism	Metabolism	0.0673343
mmu00592	alpha-Linolenic acid metabolism	Metabolism	0.1890495
mmu00830	Retinol metabolism	Metabolism	0.2058288
mmu00980	Metabolism of xenobiotics by cytochrome P450	Metabolism	0.4722742
mmu00982	Drug metabolism - cytochrome P450	Metabolism	0.4629128
mmu01040	Biosynthesis of unsaturated fatty acids	Metabolism	0.2569789
mmu02010	ABC transporters	Environmental Information Processing	0.3653957
mmu03050	Proteasome	Genetic Information Processing	0.3369527
mmu04010	MAPK signaling pathway	Environmental Information Processing	0.4725408
mmu04014	Ras signaling pathway	Environmental Information Processing	0.0522714
mmu04015	Rap1 signaling pathway	Environmental Information Processing	0.5583109
mmu04020	Calcium signaling pathway	Environmental Information Processing	0.8802017
mmu04022	cGMP-PKG signaling pathway	Environmental Information Processing	0.1898022
mmu04024	cAMP signaling pathway	Environmental Information Processing	0.1297904
mmu04060	Cytokine-cytokine receptor interaction	Environmental Information Processing	0.2458375
mmu04061	Viral protein interaction with cytokine and cytokine receptor	Environmental Information Processing	0.0480597
mmu04062	Chemokine signaling pathway	Organismal Systems	0.2303621
mmu04064	NF-kappa B signaling pathway	Environmental Information Processing	0.0129577
mmu04066	HIF-1 signaling pathway	Environmental Information Processing	0.6323194
mmu04068	FoxO signaling pathway	Environmental Information Processing	0.3151733
mmu04070	Phosphatidylinositol signaling system	Environmental Information Processing	0.569015
mmu04072	Phospholipase D signaling pathway	Environmental Information Processing	0.730284
mmu04080	Neuroactive ligand-receptor interaction	Environmental Information Processing	0.1231931
mmu04110	Cell cycle	Cellular Processes	0.0230906
mmu04114	Oocyte meiosis	Cellular Processes	0.003926
mmu04115	p53 signaling pathway	Cellular Processes	0.1291431
mmu04120	Ubiquitin mediated proteolysis	Genetic Information Processing	0.7254567
mmu04140	Autophagy - animal	Cellular Processes	0.7130122

mmu04142	Lysosome	Cellular Processes	0.6946498
mmu04144	Endocytosis	Cellular Processes	0.9051464
mmu04146	Peroxisome	Cellular Processes	0.5293171
mmu04150	mTOR signaling pathway	Environmental Information Processing	0.7487697
mmu04151	PI3K-Akt signaling pathway	Environmental Information Processing	0.0909377
mmu04152	AMPK signaling pathway	Environmental Information Processing	0.3023253
mmu04210	Apoptosis	Cellular Processes	0.331163
mmu04211	Longevity regulating pathway	Organismal Systems	0.1837515
mmu04213	Longevity regulating pathway - multiple species	Organismal Systems	0.4187179
mmu04215	Apoptosis - multiple species	Cellular Processes	0.2438581
mmu04217	Necroptosis	Cellular Processes	0.4540168
mmu04218	Cellular senescence	Cellular Processes	0.459845
mmu04260	Cardiac muscle contraction	Organismal Systems	0.1744044
mmu04261	Adrenergic signaling in cardiomyocytes	Organismal Systems	0.1535037
mmu04270	Vascular smooth muscle contraction	Organismal Systems	0.3533777
mmu04310	Wnt signaling pathway	Environmental Information Processing	0.439296
mmu04350	TGF-beta signaling pathway	Environmental Information Processing	0.5802666
mmu04360	Axon guidance	Organismal Systems	0.7970473
mmu04371	Apelin signaling pathway	Environmental Information Processing	0.7000114
mmu04510	Focal adhesion	Cellular Processes	0.8331941
mmu04512	ECM-receptor interaction	Environmental Information Processing	0.5375352
mmu04514	Cell adhesion molecules	Environmental Information Processing	0.4481539
mmu04530	Tight junction	Cellular Processes	0.4243681
mmu04550	Signaling pathways regulating pluripotency of stem cells	Cellular Processes	0.70788
mmu04610	Complement and coagulation cascades	Organismal Systems	0.0468092
mmu04613	Neutrophil extracellular trap formation	Organismal Systems	0.5427887
mmu04620	Toll-like receptor signaling pathway	Organismal Systems	0.2121926
mmu04621	NOD-like receptor signaling pathway	Organismal Systems	0.1050652
mmu04622	RIG-I-like receptor signaling pathway	Organismal Systems	0.4629128
mmu04623	Cytosolic DNA-sensing pathway	Organismal Systems	0.1379603
mmu04625	C-type lectin receptor signaling pathway	Organismal Systems	0.253934
mmu04640	Hematopoietic cell lineage	Organismal Systems	0.5535488
mmu04657	IL-17 signaling pathway	Organismal Systems	0.0012384
mmu04659	Th17 cell differentiation	Organismal Systems	0.5983784
mmu04662	B cell receptor signaling pathway	Organismal Systems	0.4994028
mmu04668	TNF signaling pathway	Environmental Information Processing	0.0175679
mmu04670	Leukocyte transendothelial migration	Organismal Systems	0.641938
mmu04713	Circadian entrainment	Organismal Systems	0.5765487
mmu04714	Thermogenesis	Organismal Systems	0.1406263
mmu04720	Long-term potentiation	Organismal Systems	0.4436957
mmu04722	Neurotrophin signaling pathway	Organismal Systems	0.2829891
mmu04723	Retrograde endocannabinoid signaling	Organismal Systems	0.3690958
mmu04725	Cholinergic synapse	Organismal Systems	0.6257657
mmu04727	GABAergic synapse	Organismal Systems	0.541591
mmu04728	Dopaminergic synapse	Organismal Systems	0.3279723
mmu04730	Long-term depression	Organismal Systems	0.4084198
mmu04740	Olfactory transduction	Organismal Systems	0.9982964
mmu04742	Taste transduction	Organismal Systems	0.1900242
mmu04744	Phototransduction	Organismal Systems	0.022694

mmu04750	Inflammatory mediator regulation of TRP channels	Organismal Systems	0.6722416
mmu04810	Regulation of actin cytoskeleton	Cellular Processes	0.8701394
mmu04910	Insulin signaling pathway	Organismal Systems	0.3407107
mmu04911	Insulin secretion	Organismal Systems	0.1713067
mmu04912	GnRH signaling pathway	Organismal Systems	0.1837515
mmu04913	Ovarian steroidogenesis	Organismal Systems	0.1036005
mmu04914	Progesterone-mediated oocyte maturation	Organismal Systems	0.0455752
mmu04915	Estrogen signaling pathway	Organismal Systems	0.1105032
mmu04916	Melanogenesis	Organismal Systems	0.2153822
mmu04917	Prolactin signaling pathway	Organismal Systems	0.1350062
mmu04918	Thyroid hormone synthesis	Organismal Systems	0.1350062
mmu04921	Oxytocin signaling pathway	Organismal Systems	0.7396887
mmu04922	Glucagon signaling pathway	Organismal Systems	0.2281839
mmu04923	Regulation of lipolysis in adipocytes	Organismal Systems	0.392634
mmu04924	Renin secretion	Organismal Systems	0.4814744
mmu04925	Aldosterone synthesis and secretion	Organismal Systems	0.221775
mmu04926	Relaxin signaling pathway	Organismal Systems	0.1014881
mmu04927	Cortisol synthesis and secretion	Organismal Systems	0.4676138
mmu04928	Parathyroid hormone synthesis, secretion and action	Organismal Systems	0.0672516
mmu04929	GnRH secretion	Organismal Systems	0.4238005
mmu04930	Type II diabetes mellitus	Human Diseases	0.3427402
mmu04931	Insulin resistance	Human Diseases	0.2474839
mmu04932	Non-alcoholic fatty liver disease	Human Diseases	0.0463691
mmu04934	Cushing syndrome	Human Diseases	0.412284
mmu04935	Growth hormone synthesis, secretion and action	Organismal Systems	0.641938
mmu04936	Alcoholic liver disease	Human Diseases	0.3407107
mmu04940	Type I diabetes mellitus	Human Diseases	0.4238005
mmu04950	Maturity onset diabetes of the young	Human Diseases	0.022694
mmu04960	Aldosterone-regulated sodium reabsorption	Organismal Systems	0.28255
mmu04962	Vasopressin-regulated water reabsorption	Organismal Systems	0.3192868
mmu04970	Salivary secretion	Organismal Systems	0.5251542
mmu04971	Gastric acid secretion	Organismal Systems	0.4814744
mmu04972	Pancreatic secretion	Organismal Systems	0.6290568
mmu04974	Protein digestion and absorption	Organismal Systems	0.6123106
mmu04975	Fat digestion and absorption	Organismal Systems	0.3072523
mmu04976	Bile secretion	Organismal Systems	0.5839523
mmu04977	Vitamin digestion and absorption	Organismal Systems	0.1890495
mmu05010	Alzheimer disease	Human Diseases	0.2385814
mmu05012	Parkinson disease	Human Diseases	0.1961302
mmu05014	Amyotrophic lateral sclerosis	Human Diseases	0.3974012
mmu05016	Huntington disease	Human Diseases	0.1213141
mmu05020	Prion disease	Human Diseases	0.2032866
mmu05022	›athways of neurodegeneration - multiple disease:	Human Diseases	0.3890337
mmu05030	Cocaine addiction	Human Diseases	0.3427402
mmu05031	Amphetamine addiction	Human Diseases	0.1204683
mmu05032	Morphine addiction	Human Diseases	0.5495975
mmu05033	Nicotine addiction	Human Diseases	0.295008
mmu05034	Alcoholism	Human Diseases	0.1023131
mmu05100	Bacterial invasion of epithelial cells	Human Diseases	0.1409288

mmu05132	Salmonella infection	Human Diseases	0.6479564
mmu05133	Pertussis	Human Diseases	0.4860151
mmu05134	Legionellosis	Human Diseases	0.4084198
mmu05145	Toxoplasmosis	Human Diseases	0.2442613
mmu05146	Amoebiasis	Human Diseases	0.2346066
mmu05150	Staphylococcus aureus infection	Human Diseases	0.2765334
mmu05152	Tuberculosis	Human Diseases	0.2031147
mmu05160	Hepatitis C	Human Diseases	0.0519759
mmu05161	Hepatitis B	Human Diseases	0.4153165
mmu05162	Measles	Human Diseases	0.7155448
mmu05163	Human cytomegalovirus infection	Human Diseases	0.6436122
mmu05164	Influenza A	Human Diseases	0.7721614
mmu05165	Human papillomavirus infection	Human Diseases	0.3695369
mmu05166	Human T-cell leukemia virus 1 infection	Human Diseases	0.6280819
mmu05167	Kaposi sarcoma-associated herpesvirus infection	Human Diseases	0.5684538
mmu05168	Herpes simplex virus 1 infection	Human Diseases	0.7568673
mmu05169	Epstein-Barr virus infection	Human Diseases	0.3056658
mmu05170	Human immunodeficiency virus 1 infection	Human Diseases	0.6073659
mmu05171	Coronavirus disease - COVID-19	Human Diseases	0.6303316
mmu05200	Pathways in cancer	Human Diseases	0.5111144
mmu05202	Transcriptional misregulation in cancer	Human Diseases	0.5659335
mmu05203	Viral carcinogenesis	Human Diseases	0.5808998
mmu05204	Chemical carcinogenesis - DNA adducts	Human Diseases	0.520955
mmu05205	Proteoglycans in cancer	Human Diseases	0.8316984
mmu05206	MicroRNAs in cancer	Human Diseases	0.7596823
mmu05207	Chemical carcinogenesis - receptor activation	Human Diseases	0.5454012
mmu05208	Chemical carcinogenesis - reactive oxygen specie	Human Diseases	0.3032793
mmu05214	Glioma	Human Diseases	0.4768943
mmu05215	Prostate cancer	Human Diseases	0.054557
mmu05221	Acute myeloid leukemia	Human Diseases	0.4581709
mmu05222	Small cell lung cancer	Human Diseases	0.0468092
mmu05225	Hepatocellular carcinoma	Human Diseases	0.7820705
mmu05226	Gastric cancer	Human Diseases	0.730284
mmu05321	Inflammatory bowel disease	Human Diseases	0.4135912
mmu05322	Systemic lupus erythematosus	Human Diseases	0.3659633
mmu05323	Rheumatoid arthritis	Human Diseases	0.5293171
mmu05412	Arrhythmogenic right ventricular cardiomyopath	Human Diseases	0.4905164
mmu05415	Diabetic cardiomyopathy	Human Diseases	0.109257
mmu05417	Lipid and atherosclerosis	Human Diseases	0.288987
mmu05418	Fluid shear stress and atherosclerosis	Human Diseases	0.7230111
